# Care Robots as Emerging Health Technologies: Systematic Review and Meta-Analysis of Randomized Controlled Trials

**DOI:** 10.2196/95232

**Published:** 2026-06-30

**Authors:** Gaeun Kim, Jinmi Jeon

**Affiliations:** 1 Department of Nursing, College of Nursing, Keimyung University, 1095, Dalgubeol-daero, Dalseo-gu, Daegu, 42601, Republic of South Korea, 82532587658; 2 Graduate School, Department of Nursing, Keimyung University, Daegu, Republic of South Korea; 3 Department of Nursing, Keimyung University Dongsan Hospital, Daegu, Republic of Korea

**Keywords:** socially assistive robots, care robots, digital health, robotics, systematic review, meta-analysis

## Abstract

**Background:**

Global aging and health care workforce shortages are increasing demand for therapeutic support among older adult and pediatric populations. Care robots, an umbrella term for socially assistive robots, companion and pet platforms, and therapeutic humanoids sharing a social interaction component, have been proposed as embodied digital health adjuncts, but prior syntheses have remained population- or platform-specific, leaving cross-population and cross-platform effects unquantified.

**Objective:**

This review quantifies the pooled effects of care-robot interventions across 7 prespecified outcome domains, examines robot platform, target population, and intervention-duration moderators, and grades evidence certainty.

**Methods:**

Following PRISMA (Preferred Reporting Items for Systematic Reviews and Meta-Analyses) 2020, we searched 5 databases (PubMed, including MEDLINE, Embase, Cochrane CENTRAL, CINAHL, APA PsycINFO) and 2 trial registries (ClinicalTrials.gov; World Health Organization International Clinical Trials Registry Platform) through April 29, 2026, with no language restriction. Eligible studies were randomized controlled trials of embodied care robots versus standard care, active controls, or waitlist. Random-effects meta-analysis used Hedges *g* with Hartung-Knapp-Sidik-Jonkman correction; for domains with k≥4, we report 95% CIs and prediction intervals (PIs). Risk of bias used the Cochrane Risk of Bias 2 tool; certainty used the Grading of Recommendations Assessment, Development and Evaluation (GRADE) framework.

**Results:**

A total of 34 randomized controlled studies (n=2476; 17 countries; 2015‐2024) met inclusion criteria; 20 contributed to at least 1 meta-analysis, and 14 entered narrative synthesis. Under Hartung-Knapp-Sidik-Jonkman pooling, only neuropsychiatric symptoms reached significance: Hedges *g*=0.44 (95% CI 0.03-0.84; 95% PI −0.42 to 1.30; k=6; *P*=.04). The remaining 6 domains were favorable but nonsignificant: quality of life Hedges *g*=0.15 (95% CI −0.41 to 0.71; 95% PI −0.86 to 1.16; k=5); depression Hedges *g*=0.20 (95% CI −0.08 to 0.49; 95% PI −0.36 to 0.76; k=7); agitation Hedges *g*=0.32 (95% CI −0.07 to 0.71; 95% PI −0.26 to 0.89; k=4); stress and pain Hedges *g*=0.53 (95% CI −0.48 to 1.53; 95% PI −1.57 to 2.62; k=6); social-communicative skills Hedges *g*=0.45 (95% CI −0.52 to 1.42; 95% PI −1.66 to 2.56; k=5); and cognitive function Hedges *g*=0.18 (95% CI −0.62 to 0.98; 95% PI −1.11 to 1.47; k=4). All 7 PIs encompassed the null, indicating no assured effect in new settings. The neuropsychiatric symptom result was fragile: significance was lost in 5 of 6 leave-one-out iterations and on excluding Petersen 2016 (data extraction ambiguity), yielding a Hedges *g* of 0.40 (*P*=.10). GRADE certainty was low for neuropsychiatric symptoms and very low for the remaining 6 domains.

**Conclusions:**

Across cross-population, cross-platform randomized controlled trial pooling, the evidence does not yet support routine clinical adoption of care robots. Potential benefits are narrow, of low certainty, and contingent on adequately powered multicenter confirmatory trials with PI-informed sample sizes. Care robots are best framed as facilitator-supported adjuncts that augment, rather than substitute for, human-delivered care.

## Introduction

Health care systems worldwide face 2 parallel and increasingly convergent pressures that are reshaping how direct patient care is organized. The first is demographic: by 2050, the global population aged 60 years and older is projected to reach 2.1 billion, with corresponding rises in the prevalence of chronic disease, cognitive impairment, and dementia [1]. The second is structural: a projected deficit of 12.9 million health care professionals by 2035 [2] is already constraining the dose and continuity of one-to-one, relational care, most acutely in long-term care and in specialized pediatric therapy. Beyond these aggregate workforce figures, specific populations—children with autism spectrum disorder (ASD) and other neurodevelopmental conditions, pediatric inpatients undergoing anxiety-provoking procedures, and older adults with dementia in residential care—share a common practical problem: they benefit from structured, predictable, attention-capturing human interaction that is difficult to deliver at scale.

Care robots have emerged as one technological response to this shared problem. They are physically embodied platforms designed for direct patient interaction and include socially assistive robots such as PARO, NAO, Pepper, Kabochan, and Kaspar; companion and pet robots such as Joy for All, iRobiQ, and MiRo; and specialized therapeutic humanoids [3]. Unlike disembodied digital health tools—telehealth platforms, mobile health apps, or wearable sensors—care robots offer multimodal physical presence (tactile, visual, auditory, and increasingly conversational) that may elicit social and emotional responses in ways that screen-based interventions do not [4]. In older adults with cognitive impairment, tangible tactile interaction with companion robots is thought to support emotional regulation and person-centered engagement in a manner consistent with Kitwood’s model of dementia care [5]. In children with ASD, the simplified, predictable, and repeatable social behavior of humanoid robots has been proposed as a scaffold for practicing social-communicative skills that children may find difficult to rehearse with human partners [6]. Acceptance and usability—rather than efficacy alone—are recognized as critical determinants of whether such platforms translate from trials into practice [7,8]. Beyond stand-alone use, socially assistive robots are increasingly deployed as components of broader technological ecosystems that integrate sensing, data capture, and connected services rather than as isolated devices [9,10].

Despite this shared theoretical rationale, the empirical literature on care robots has developed in siloed streams. Prior systematic reviews and meta-analyses have either restricted themselves to a single population—Pu et al [11] in older adults, Leng et al [12] in dementia care with pet robots, and Yu et al [13] in dementia broadly; or Scassellati et al [6] in children with ASD—or to a single platform, most commonly PARO in dementia care. The collective consequence is that the most clinically actionable question—whether observed robot-intervention effects are driven primarily by platform (eg, PARO vs humanoid) or by population (eg, dementia vs ASD vs pediatric procedural anxiety)—cannot be answered from any one of these reviews alone, because each review holds either platform or population fixed by design. Two further consequences follow for practice. First, procurement, deployment, and health technology assessment decisions are increasingly made across populations (eg, a long-term care operator deciding among PARO, NAO, and companion pet platforms for mixed-acuity settings), yet there is no published synthesis that places the platform effects observed in one population against those observed in another within a single analytic framework. Second, each single-population review has necessarily treated implementation questions—facilitator training, acceptance, setting fit—in isolation, missing the opportunity to identify cross-cutting patterns. Because care-robot randomized controlled trials (RCTs) have grown substantially over the past decade (2015‐2024), the minimum number of studies (k) required for meaningful moderation analyses of robot platform and target population is now within reach.

Accordingly, this systematic review and meta-analysis had 4 prespecified objectives. First, to quantify the pooled effects of care-robot interventions across 7 prespecified outcome domains in a cross-population sample that explicitly includes both older adult and pediatric populations. Second, to examine the moderating effects of robot platform (PARO vs humanoid vs other companion and pet), target population (dementia or older adult vs pediatric or ASD vs other), and intervention duration (≥10 weeks vs <10 weeks) through prespecified subgroup analyses. Third, to appraise the methodological quality of included studies using the Cochrane Risk of Bias 2 (RoB 2) tool. Fourth, to evaluate the certainty of evidence across domains using the Grading of Recommendations Assessment, Development and Evaluation (GRADE) framework and to translate the resulting evidence profile into realistic, targeted recommendations for clinical use. To our knowledge, this is among the first quantitative syntheses to pool care-robot RCTs across both older adult and pediatric populations within a common framework; because per-subgroup study counts are small, we frame the resulting comparison across robot platform and populations as exploratory and hypothesis-generating rather than confirmatory.

## Methods

### Overview

This systematic review and meta-analysis is reported in accordance with the PRISMA (Preferred Reporting Items for Systematic Reviews and Meta-Analyses) 2020 Statement [14], the PRISMA 2020 for Abstracts extension, the PRISMA-S (Preferred Reporting Items for Systematic Reviews and Meta-Analyses literature search) extension for reporting literature searches [15], and the methodological standards of the *Cochrane Handbook for Systematic Reviews of Interventions* [16]. Completed PRISMA 2020 Expanded, PRISMA 2020 for Abstracts, and PRISMA-S checklists are provided as Checklist1^3^. The review was not prospectively registered in PROSPERO (International Prospective Register of Systematic Reviews); an a priori review protocol specifying eligibility criteria, outcome domains, analytic plan, and risk-of-bias assessment was developed and dated before the formal search and is available from the corresponding author on reasonable request.

### Eligibility Criteria

Eligibility criteria were defined using the Population, Intervention, Comparison, Outcomes, and Study Design framework. The population comprised patients of any age, in any clinical or community setting, with no restrictions on primary diagnosis or age group, consistent with the cross-population aim of the review. The intervention was any physically embodied care robot designed for direct patient interaction, including socially assistive, companion, pet, and therapeutic humanoid platforms. Because the eligibility criteria required a social or interactive component, all included platforms are, by definition, social robots or socially assistive robots; we retain “care robots” as the umbrella term while noting that every included device met the social robots or socially assistive robots criterion. Surgical robots, passive rehabilitation exoskeletons without a social interaction component, and clinician-controlled telepresence robots were excluded. Comparators were standard care without robot interaction, attention-matched active controls (eg, reading or tablet-based sessions of equivalent duration), placebo conditions (deactivated robot or plush equivalent), and waitlist controls. Outcomes were restricted to quantitative, validated patient-reported outcome measures within 7 prespecified outcome domains defined in the a priori protocol: neuropsychiatric symptoms (NPS), quality of life, depression, agitation, stress and pain, social-communicative skills, and cognitive function. Other outcomes encountered during extraction (eg, motor activity and biomarkers) were retained in the narrative synthesis but not pooled. Eligible study designs were individual RCTs and cluster RCTs. Single-arm before-after studies, case reports, qualitative-only studies, feasibility studies without an outcome comparator, and conference abstracts lacking full numerical data were excluded.

### Information Sources

We searched 5 electronic databases: PubMed (MEDLINE), Embase (Elsevier), the Cochrane Central Register of Controlled Trials (CENTRAL), CINAHL Complete (EBSCO), and APA PsycINFO. Two trial registries (ClinicalTrials.gov and the World Health Organization [WHO] International Clinical Trials Registry Platform [ICTRP]) were searched for completed trials with reported results. Reference lists of all included studies and of 4 prior systematic reviews in the field were screened, and forward citation tracking was performed via Google Scholar. Searches covered database inception through April 29, 2026. No language or publication date restrictions were applied at the search stage. At full-text screening, articles for which no English or Korean full text or professional translation was obtainable despite repeated retrieval attempts were excluded (n=5); this is reported in the PRISMA flow diagram.

### Search Strategy

Search strategies were developed iteratively in consultation with a health-sciences librarian and combined controlled vocabulary (MeSH [Medical Subject Headings] terms in PubMed and CENTRAL; EMTREE terms in Embase; CINAHL Subject Headings; APA Thesaurus terms in PsycINFO) with free-text terms mapped with field modifiers (.ti,ab,kw and database-specific equivalents), truncation, and proximity operators. Three concept blocks were combined with AND: (1) care robots, including both generic terms (“social robot,” “socially assistive robot,” “companion robot,” “robotic pet,” “humanoid robot,” and “therapeutic robot”) and platform-specific trade names (PARO, NAO, Pepper, Kaspar, Kabochan, CommU, Joy for All, MiRo, and iRobiQ); (2) health care context terms (eg, “nursing home,” “long-term care,” “dementia care,” “pediatric,” “hospital,” “rehabilitation,” and “outpatient”); and (3) patient-outcome terms covering the 7 prespecified domains (eg, “quality of life,” “neuropsychiatric symptoms,” “agitation,” “anxiety,” “pain,” “social skills,” and “mental status”). Full database-specific search strategies, exact search dates, and the number of records retrieved per database are reported in Checklist 1 (PRISMA-S compliant).

### Selection Process

All retrieved records were imported into Covidence systematic review software (Veritas Health Innovation) and deduplicated automatically with manual verification. Two reviewers (GK and JJ) independently screened all unique records by title and abstract and then by full text against the prespecified eligibility criteria. Both reviewers are registered nurses: GK holds a PhD and has prior published systematic review experience in gerontological and digital health domains and completed formal training in the Cochrane RoB 2 and GRADE frameworks; JJ holds a master’s degree and completed Cochrane-endorsed methodological training in systematic review conduct and data extraction. Interrater reliability was quantified using Cohen kappa; Cohen κ=0.89 at title and abstract and Cohen κ=0.92 at full text [17]. Disagreements were resolved through structured discussion; residual disagreements were adjudicated by a third senior reviewer with prior systematic review and meta-analysis experience.

### Data Collection Process and Data Items

A standardized data extraction form was piloted on 5 [18-22] randomly selected studies and refined before full-scale extraction. Two reviewers independently extracted: study identification (first author, year, country, and journal); study design and randomization method; participant characteristics (sample size per arm, mean age, sex distribution, primary diagnosis, and care setting); intervention details (robot type and model, session duration and frequency, total intervention duration, presence and role of facilitator, group vs individual format); control condition (standard care, attention-matched active control, placebo or deactivated robot, and waitlist); outcome measures with instrument names, assessment time points, and numerical data (means, SDs, and sample sizes); and adverse events or dropouts. For studies reporting outcome data only in graphical form, values were extracted using WebPlotDigitizer (version 4.6; Ankit Rohatgi). Discrepancies were resolved by consensus. Where standard deviations were not directly reported, they were imputed from 95% CIs, SEs, or IQRs using Cochrane Handbook–recommended formulas [16]. Studies for which sufficient numerical data could not be obtained were excluded from meta-analyses but retained in the narrative synthesis.

Robot platforms were classified a priori into 3 operationally defined categories for the subgroup analyses: (1) PARO and PARO-like tactile companion robots—biofeedback-responsive, nonverbal, animal-form-factor platforms whose interaction modality is primarily tactile; (2) humanoid robots—bipedal or upper-body humanoid form factor with structured verbal and gestural interaction (NAO, Pepper, Kaspar, CommU, and Kabochan); and (3) other companion and pet and nonhumanoid platforms (Joy for All, iRobiQ, MiRo, and unnamed custom platforms).

### Study Risk-of-Bias Assessment

The methodological quality of all included randomized and cluster-randomized trials was assessed independently by 2 reviewers using the Cochrane RoB 2) tool [23] across 5 domains: (1) bias arising from the randomization process, (2) bias due to deviations from intended interventions, (3) bias due to missing outcome data, (4) bias in measurement of the outcome, and (5) bias in selection of the reported result. As specified in the Multimedia Appendix 1 footnote, all 34 included studies [18-22,24-52] were classified as randomized controlled studies (24 individual RCTs [19-22,31,32,34-51] and 10 [18,24-30,33,52] cluster RCTs) following revision-process reclassification and were therefore assessed using a single risk-of-bias instrument (RoB 2). Overall study risk was classified as low, some concerns, or high. Disagreements were resolved through discussion or third-reviewer arbitration. Domain-level and overall judgments were used to inform GRADE certainty downgrading for risk of bias.

### Effect Measures and Synthesis Methods

Given heterogeneous outcome instruments across included studies, effect sizes were expressed as standardized mean differences (Hedges *g*) with 95% CIs. Hedges *g* was preferred over Cohen *d* for its reduced small-sample bias [53]. Random-effects meta-analysis was performed using the restricted maximum-likelihood estimator for between-study variance τ² with the Hartung-Knapp-Sidik-Jonkman (HKSJ) correction for the CI of the pooled estimate [54,55]. The HKSJ correction was selected, in place of the DerSimonian-Laird method used in our original submission, because it provides more reliable CI coverage and reduces false-positive rates under small-study and moderate-to-high heterogeneity conditions [55]. All effect sizes were oriented so that a positive Hedges *g* indicates an effect favoring care robots across all domains; for symptom reduction outcomes (NPS, agitation, depression, and stress and pain), change scores were sign-reversed accordingly. Effect sizes were interpreted using Cohen conventional benchmarks: small (|*g*|=0.20‐0.49), moderate (|*g*|=0.50‐0.79), and large (|*g*|≥0.80) [53].

Statistical heterogeneity was quantified using *I*², the between-study variance τ², and the Cochran Q test. For each domain with k≥4, we additionally calculated the 95% prediction interval (PI) [56,57], which describes the range within which the true effect in a new setting is expected to fall and therefore reflects the real-world implications of between-study heterogeneity more faithfully than *I*² alone [56]. Where the PI crosses the line of no effect despite a statistically significant CI for the pooled estimate, we interpret this as indicating that, although an average benefit is detectable across existing studies, the effect in a new setting cannot be assumed to be beneficial.

To address the possibility of double-counting, participant cohorts that appeared across multiple publications were identified a priori. We applied the following principle: each cohort contributes to each outcome pool at most once, but the same cohort may contribute to different outcome pools when it measures different outcome domains, because each outcome is analyzed in an independent meta-analysis [58]. Four cohort-overlap clusters were identified: (1) Australian PARO cluster (shared n=415)—Moyle et al [24], Moyle et al [25], Jones et al [26], and Mervin et al [27] drew from a shared dementia cohort; only Moyle et al [24] was retained as the primary publication contributing to the NPS and agitation pools, while Moyle et al [25], Jones et al [26], and Mervin et al [27] are narrative-only. (2) Norwegian PARO cluster—Jøranson et al [28], Jøranson et al [29], and Jøranson et al [30] drew from a shared nursing-home dementia cohort; Jøranson et al [28] was retained for the NPS, depression, and agitation pools; Jøranson et al [29] was retained for the quality-of-life pool (a different outcome domain measured in the same cohort), and Jøranson et al [30] is narrative-only. (3) Hong Kong dementia cohort—Chen et al [18] and Chen et al [31] drew from a shared cohort; Chen et al [18] was retained as the primary publication contributing to the relevant outcome pools, while Chen et al [31] (a technology-acceptance secondary analysis of the same cohort) is narrative-only. (4) Dutch ASD cohort (n=81)—De Korte et al [19] and van den Berk-Smeekens et al [32] drew from a shared trial; only De Korte et al [19] was retained for the social-communicative skills pool, as van den Berk-Smeekens et al [32] reported a secondary analysis of the same trial. Studies measuring multiple outcomes (eg, Valentí-Soler et al [33] contributing to 5 outcome pools and Chen et al [18], Petersen et al [34], and Pollak et al [20] each contributing to 4 outcome pools) were retained in each relevant pool, with each contribution representing an independent outcome estimate from a single cohort. As a result, no cohort contributes to the same pooled estimate more than once. The splitting approach (retaining one publication per cohort per outcome pool) was selected over multilevel or robust-variance-estimation alternatives because the within-cohort correlation across the identified clusters could not be reliably estimated from the available aggregate data. This allocation is documented in a footnote to Multimedia Appendix 1.

Prespecified subgroup analyses were conducted by (1) robot platform (PARO vs humanoid vs other companion and pet), (2) target population (dementia or older adult vs pediatric or ASD vs other), and (3) intervention duration (≥10 weeks vs <10 weeks). Sensitivity analyses comprised: (1) leave-one-out (LOO) analysis (one study excluded at a time, applied to any pool reaching statistical significance under primary HKSJ pooling); (2) exclusion of studies rated overall high risk of bias under RoB 2 (4 of 34 studies); and (3) data extraction sensitivity analysis (exclusion of Petersen 2016, flagged for directional ambiguity in published change-score reporting). All meta-analyses were conducted in R (version 4.3.2; R Core Team) using the meta (version 6.5‐0; Guido Schwarzer) and metafor (version 4.4‐0; Wolfgang Viechtbauer) packages, with metafor::rma(yi, vi, method=

“REML,” test=“knha”) for the HKSJ-corrected pooled estimate and predict for the 95% PI.

### Reporting Bias Assessment

We assessed small-study effects, rather than publication bias per se, using visual inspection of contour-enhanced funnel plots and the Egger weighted-regression test [59]. Following Sterne et al [60], we note that funnel-plot asymmetry can arise from publication bias but also from true effect-size differences between small and large studies, methodological differences between small and large studies, or chance. The Egger test was interpreted only for domains with k≥10 contributing studies because the test has very limited power below that threshold; nonsignificant Egger results in smaller domains should not be read as ruling out small-study effects. Given that no domain in our review reached k≥10, the funnel plots reported for NPS (k=6) and depression (k=7) are presented for transparency only and are explicitly interpreted with caution.

### Certainty Assessment

Certainty of evidence for each primary outcome domain was evaluated independently by 2 reviewers using the GRADE framework [61], with downgrading for risk of bias, inconsistency, indirectness, imprecision, and reporting biases. The formal GRADE evidence profile was produced using GRADEpro GDT (McMaster University).

### Ethical Considerations

As this study is a systematic review and meta-analysis of previously published aggregate data, institutional review board approval was not required, and no individual participant data were accessed.

## Results

### Study Selection

**Figure 1.**
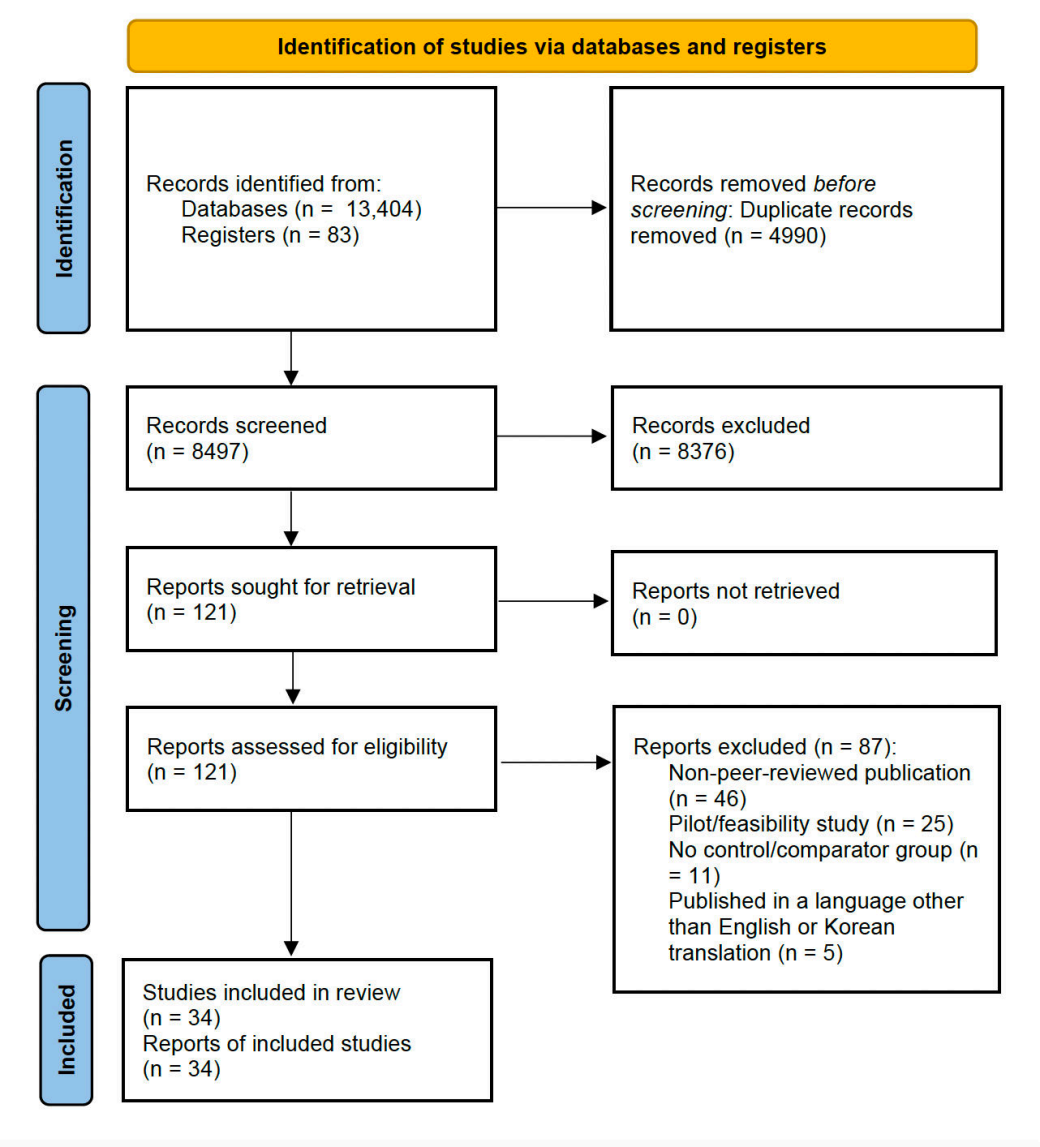
PRISMA (Preferred Reporting Items for Systematic Reviews and Meta-Analyses) 2020 flow diagram for the systematic review and meta-analysis of care robot interventions. Of the 34 included trials, 20 contributed to ≥1 meta-analysis pool and 14 entered narrative-only synthesis. Adapted from Page et al [14]. EN: English; KR: Korean.

The expanded search across 5 databases and 2 trial registries yielded 13,487 records (PubMed, including MEDLINE n=5935; Embase n=4034; Cochrane CENTRAL n=2258; CINAHL n=1176; APA PsycINFO n=1; ClinicalTrials.gov n=75; WHO ICTRP n=8). APA PsycINFO returned only 1 record matching all 3 concept blocks; relaxed-vocabulary searches in PsycINFO yielded substantially more records but with high duplication against MEDLINE and Embase and were excluded to avoid double-counting. After deduplication in EndNote 21 with manual verification in Covidence, 4990 duplicates were removed and 8497 unique records underwent title-and-abstract screening, of which 8376 were excluded as clearly ineligible. Full-text assessment was conducted for 121 records; 87 were excluded for the following reasons: not peer-reviewed, n=46; pilot study without a comparative arm, n=25; nonrandomized study design (single-arm before-after or noncontrolled), n=6; no English or Korean full text or professional translation obtainable, n=5; and no concurrent control group, n=5. A final total of 34 studies [18-22,24-52] met all eligibility criteria and were included in the qualitative synthesis; 20 [18-22,24,28,29,33,34,36,38,39,42,43,45-47,50,52] contributed to at least one meta-analysis pool and 14 [25-27,30-32,35,37,40,41,44,48,49,51] entered narrative-only synthesis. The updated PRISMA 2020 flow diagram is presented in Figure 1.

We updated and expanded the search strategy in accordance with Cochrane guidance. In addition to PubMed, including MEDLINE, we searched Embase, Cochrane CENTRAL, CINAHL, and APA PsycINFO to capture evidence across medicine, nursing, and behavioral psychology. We also searched ClinicalTrials.gov and the WHO ICTRP separately for ongoing or unpublished trials. Google Scholar was searched as a supplementary source for gray literature; the first 200 results for the principal search query were screened by title and abstract. Reference lists of included studies and relevant prior systematic reviews were hand-searched. No language restriction was applied at the search stage. Full database-specific search strategies, including controlled vocabulary and free-text terms, are provided in Checklist 1.

### Study Characteristics

The 34 [18-22,24-52] included studies were conducted across 17 countries and published between 2015 and 2024. Countries with the greatest representation included Australia (n=4), United States (n=4), Norway (n=3), Netherlands (n=3), and Hong Kong (n=3), followed by Greece, Israel, Italy, New Zealand, and the United Kingdom (n=2 each), and one study each from Austria, Canada, Germany, Japan, South Korea, Spain, and Taiwan. Study designs comprised randomized controlled studies in all 34 included reports—24 (70.6%) individual RCTs and 10 (29.4%) cluster RCTs; on reexamination of the original full-text sources, 5 [33,35,41,42,48] studies that were previously classified as quasi-experimental, controlled trial, or clinical trial were reclassified as randomized trials, and Valentí-Soler et al [33] was reclassified as randomized for the nursing-home arm with the sequential day care center phase noted in Limitations. Robot platforms included PARO (11/34, 32.4%), humanoid platforms (NAO, Pepper, Kabochan, CommU, Kaspar, and unnamed; 16/34, 47.1%), and other companion and pet platforms (7/34, 20.6%). Target populations included older adults with dementia or mild cognitive impairment (14/34, 41.2%), children and adolescents with ASD (9/34, 26.5%), hospitalized or procedural pediatric patients (4/34, 11.8%), and other adults (poststroke, mixed; 7/34, 20.6%). Intervention durations were single-session (9/34, 26.5%), <4 weeks (2/34, 5.9%), 4‐9 weeks (4/34, 11.8%), and ≥10 weeks (19/34, 55.9%). Care settings comprised nursing home or long-term care (n=14), hospital (n=16), community or home (n=2), and rehabilitation (n=2). Full characteristics are in Multimedia Appendix 1 (full per-study table) and Table 1 (summary).

**Table 1.** Summary characteristics of the included care robot randomized trials. Aggregated counts and proportions are shown by study design, population, robot platform, care setting, intervention duration, and meta-analysis contribution status.

Characteristic	Values, n (%)	Notes
Study design		
Randomized controlled studies	34 (100)	—^a^
Individual RCT^b^	24 (70.6)	—
Cluster RCT	10 (29.4)	—
Robot platform		
PARO (seal-shaped SAR^c^)	11 (32.4)	Primarily dementia or LTC^d^ populations
Humanoid (NAO, Pepper, Kabochan, CommU, unnamed)	16 (47.1)	ASD^e^, pediatric, mixed populations
Companion and pet robots (Joy for All, MiRo, iRobiQ, others)	7 (20.6)	Pediatric, older adult, mixed populations
Target population		
Older adults with dementia or MCI^f^	14 (41.2)	Age range 50‐108 years
Children or adolescents with ASD	9 (26.5)	Age range 3‐12 years
Hospitalized or procedural pediatric	4 (11.7)	Acute care settings
Other adults (poststroke, mixed)	7 (20.6)	Various diagnoses
Intervention duration		
Single session	9 (26.5)	—
<4 weeks	2 (5.9)	—
4‐9 weeks	4 (11.7)	—
≥10 weeks	19 (55.9)	—
Care setting		
Nursing home or long-term care facility	14 (41.2)	—
Hospital (inpatient or outpatient)	16 (47.1)	—
Community or home setting	2 (5.9)	—
Rehabilitation center	2 (5.9)	—
Risk of bias tool		
Cochrane Risk of Bias 2 (RoB 2)	34 (100)	—

a Not applicable.

b RCT: randomized controlled trial.

c SAR: socially assistive robot.

d LTC: long-term care.

e ASD: autism spectrum disorder.

f MCI: mild cognitive impairment.

### Risk-of-Bias in Studies

**Figure 2.**
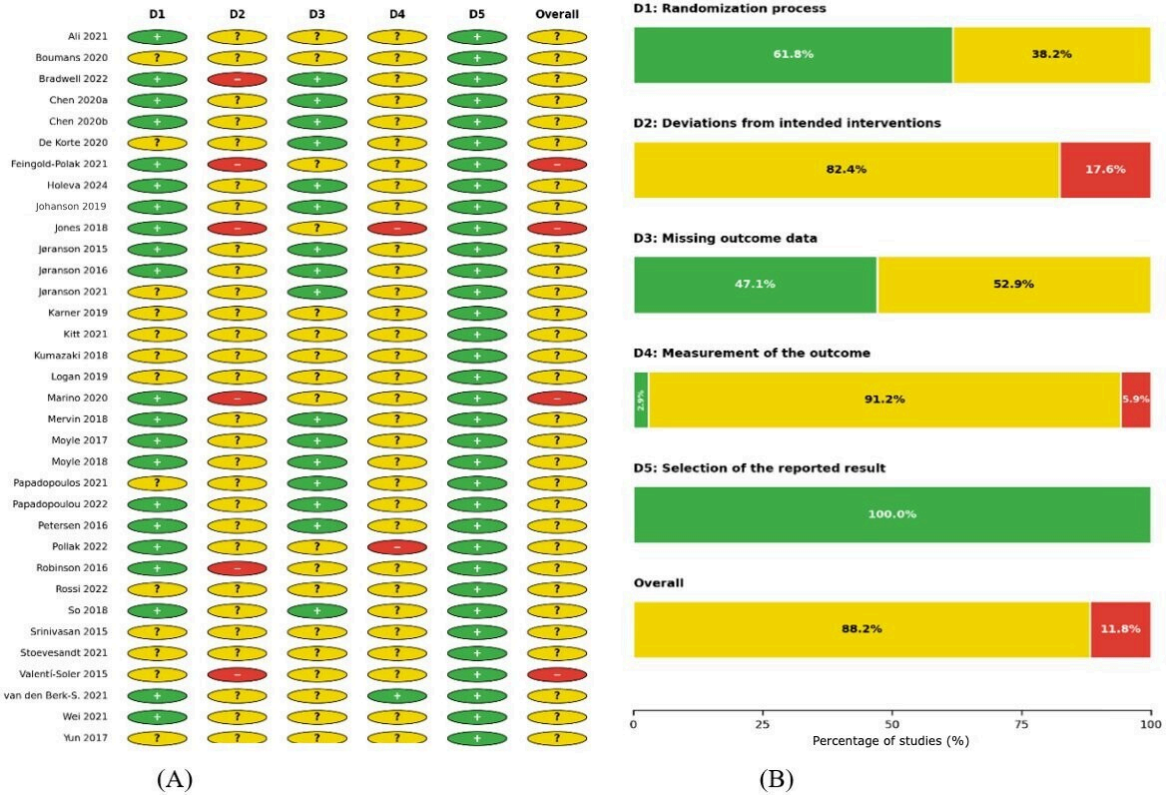
Risk-of-bias assessment of the 34 included trials using the Cochrane RoB 2 tool. (A) Study-level judgments across the 5 RoB 2 domains (D1-D5). (B) Domain-level summary across all 34 studies. Green (+) indicates low risk, yellow (?) indicates some concerns, and red (−) indicates high risk [18-22,24-52].

All 34 included randomized and cluster randomized trials were assessed using Cochrane RoB 2. Pooled across the 34 [18-22,24-52] studies, 0 studies were rated overall low risk of bias, 30 [18-22,24,25,27-32,34,36-40,42-52] (88.2%) were rated as having some concerns, and 4 [26,33,35,41] (11.8%) were rated overall high risk of bias. Performance bias (RoB 2 Domain 2: deviations from intended interventions) was the most consistently problematic domain—blinding of participants and personnel to robotic versus nonrobotic interactions is structurally impossible—and contributed to the GRADE risk-of-bias downgrade across all 7 outcome domains. Domain 5 (selective outcome reporting) was rated low risk in all studies. Missing outcome data (Domain 3) was rated low risk in 16 [18,19,24,25,27-31,34,36,42,43,46,52] (47.1%) of 34 studies and some concerns in 18 [20-22,26,32,33,35,38-41,44,45,47-51] (52.9%) studies, with no studies rated high risk on this domain. Full domain-level RoB 2 ratings are presented in Figure 2.

### Results of Syntheses

#### Overview

**Figure 3.**
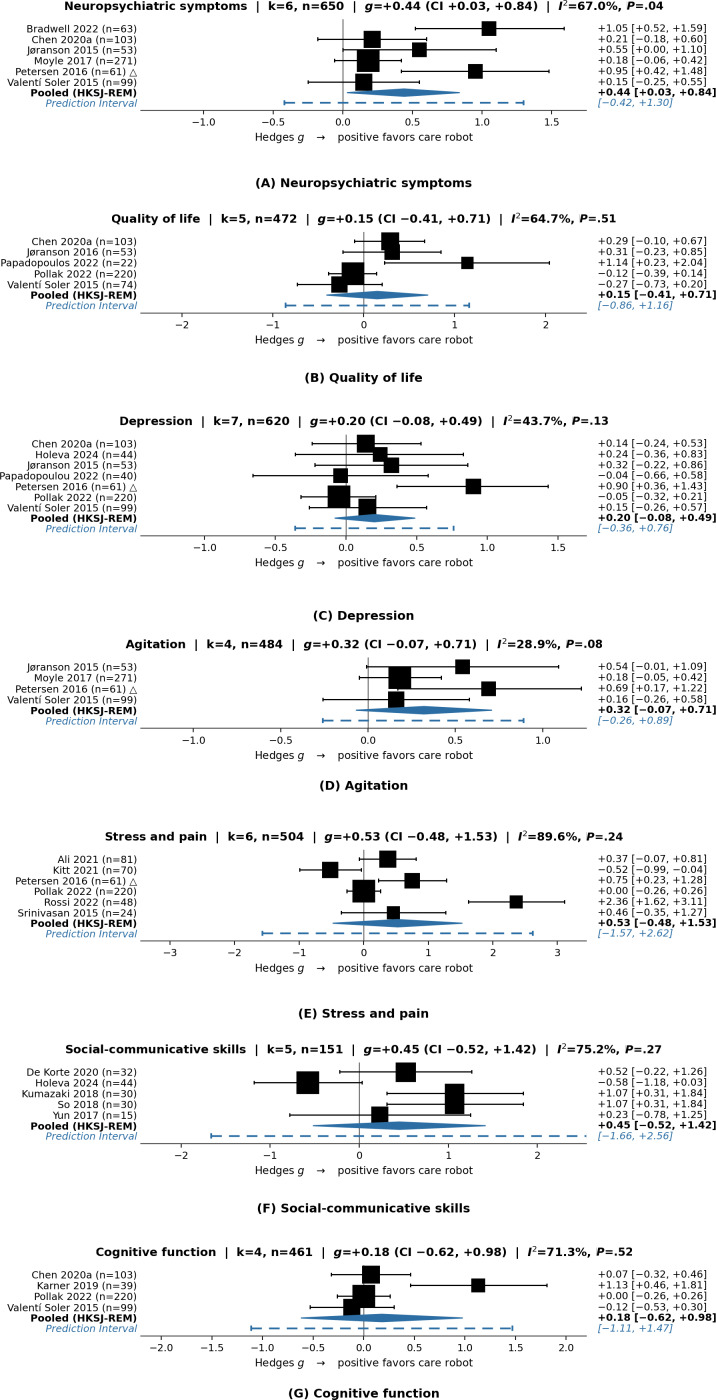
Forest plots for the 7 prespecified outcome domains. Forest plots are based on the 20 trials contributing to at least one meta-analysis. Squares indicate study-level estimates, diamonds indicate pooled HKSJ estimates, and dashed bars indicate 95% prediction intervals. Panel headers report k, N, Hedges *g*, 95% CI, *I*², and *P* value [18-22,24,28,29,33,34,36,38,39,42,45-47,50].

Following systematic full-text data verification, 20 [18-22,24,28,29,33,34,36,38,39,42,43,45-47,50,52] unique studies contributed to one or more meta-analysis pools. Pooled HKSJ random-effects estimates, 95% CIs, 95% PIs, *I*², τ², and GRADE certainty for the 7 outcome domains are summarized in Table 2, and forest plots are presented in Figure 3.

**Table 2.** GRADE^a^ summary of findings for robot-assisted interventions across 7 outcome domains^b^.

	Certainty assessment							Number of patients (number of studies)	Hedges *g* (95% CI)	Certainty	Importance
	Studies, n	Study design	Risk of bias	Inconsistency	Indirectness	Imprecision	Other considerations				
Neuropsychiatric symptoms (assessed with: NPI^c^, NPI-NH^d^, and BEHAVE-AD^e^)	6	Randomized trials	Serious^f^	Serious^g^	Not serious	Not serious	None	650 (k=6)	0.44 (CI 0.03 to 0.84)	⨁⨁◯◯ Low^f^^,^^g^	IMPORTANT
Quality of life (assessed with: QoL-AD^h^, WHOQOL^i^, or related scales)	5	Randomized trials	Serious^j^	Serious^k^	Not serious	Serious^l^	None	472 (k=5)	0.15 (–0.41 to 0.71)	⨁◯◯◯ Very low^j^^,^^k^^,^^l^	CRITICAL
Depression (assessed with: GDS^m^, CSDD^n^, PHQ-9^o^, or related scales)	7	Randomized trials	Serious^p^	Serious^q^	Not serious	Serious^r^	None	620 (k=7)	0.2 (–0.08 to 0.49)	⨁◯◯◯ Very low^p^^,^^q^^,r^	IMPORTANT
Agitation (assessed with: CMAI^s^ or related agitation scales)	4	Randomized trials	Serious^t^	Serious^u^	Not serious	Serious^v^	None	484 (k=4)	*g*0.32 (–0.07 to 0.71)	⨁◯◯◯ Very low^t^^,^^u^^,^^v^	IMPORTANT
Stress and pain (assessed with: stress scales, pain scales, or physiological indicators)	6	Randomized trials	Serious^w^	Very serious^x^	Not serious	Serious^y^	None	504 (k=6)	0.53 (–0.48 to 1.53)	⨁◯◯◯ Very low^w^^,^^x^^,^^y^	IMPORTANT
Social-communicative skills (assessed with: SRS^z^, social interaction, or communication-related scales)	5	Randomized trials	Serious^aa^	Not serious	Serious^ab^	Serious^ac^	None	151 (k=5)	0.45 (–0.52 to 1.42)	⨁◯◯◯ Very low^aa^^,^^ab^^,^^ac^	IMPORTANT
Cognitive function (assessed with: MMSE^ad^, MoCA^ae^, or related cognitive scales)	4	Randomized trials	Serious^af^	Not serious	Serious^ag^	Serious^ah^	None	461 (k=4)	0.18 (–0.62 to 0.98)	⨁◯◯◯ Very low^af^^,^^ag^^,^^ah^	CRITICAL

a GRADE: Grading of Recommendations Assessment, Development and Evaluation.

b Effect sizes are reported as Hedges *g* with 95% CIs using Hartung-Knapp-Sidik-Jonkman random-effects pooling with restricted maximum-likelihood τ² estimation. Certainty ratings were defined as ⊕⊕⊕⊕ high, ⊕⊕⊕○ moderate, ⊕⊕○○ low, and ⊕○○○ very low.

c NPI: Neuropsychiatric Inventory.

d NPI-NH: Neuropsychiatric Inventory–Nursing Home version.

e BEHAVE-AD: Behavioral Pathology in Alzheimer’s Disease Rating Scale.

f Downgraded for risk of bias due to open-label designs and concerns related to blinding, attrition, or selective reporting in some trials.

g Downgraded for inconsistency due to heterogeneity across populations, robot types, and outcome instruments.

h QoL-AD: Quality of Life in Alzheimer’s Disease scale.

i WHOQOL: World Health Organization Quality of Life.

j Downgraded for risk of bias due to open-label designs and methodological limitations in some trials.

k Downgraded for inconsistency due to heterogeneity across studies

l Downgraded for imprecision because the confidence interval crossed the null effect.

m GDS: Geriatric Depression Scale.

n CSDD: Cornell Scale for Depression in Dementia.

o PHQ-9: Patient Health Questionnaire-9.

p Downgraded for risk of bias due to methodological limitations in some included trials.

q Downgraded for inconsistency across studies.

r Downgraded for imprecision because the confidence interval crossed the null effect.

s CMAI: Cohen-Mansfield Agitation Inventory.

t Downgraded for imprecision because the confidence interval crossed the null effect.

u Downgraded for inconsistency because one study was treated as narrative-only due to cohort overlap, and the pooled evidence was limited.

v Downgraded for imprecision because the confidence interval crossed the null effect.

w Downgraded for risk of bias due to methodological limitations in included trials.

x Downgraded for severe inconsistency across studies.

y Downgraded for imprecision because the confidence interval was wide and crossed the null effect.

z SRS: Social Responsiveness Scale.

aa Downgraded for risk of bias due to open-label designs and measurement limitations.

ab Downgraded for indirectness because all studies were conducted in pediatric autism spectrum disorder populations using humanoid platforms.

ac Downgraded for imprecision because the confidence interval was wide and crossed the null effect.

ad MMSE: Mini-Mental State Examination.

ae MoCA: Montreal Cognitive Assessment.

af Downgraded for risk of bias due to methodological limitations in included trials.

ag Downgraded for indirectness because the evidence was derived from dementia and mild cognitive impairment populations.

ah Downgraded for imprecision because the confidence interval was wide and crossed the null effect.

Only NPS reached statistical significance under HKSJ random-effects pooling (Hedges *g*=0.44, 95% CI 0.03-0.84; *P*=.04). The remaining 6 domains yielded point estimates that generally favored care robots, but their 95% CIs crossed the null and did not reach conventional statistical significance. All 7 95% PIs encompassed the null, indicating that effect estimates may not generalize uniformly to new populations or settings. This conservative pattern reflects the HKSJ small-sample correction, full-text primary-source data verification, and consistent application of the Senn [58] cohort-overlap principle. Between-study heterogeneity ranged from low (*I*²=29% for agitation) to high (*I*²=90% for stress and pain).

#### NPS

Six studies (n=650) contributed measurements using the Neuropsychiatric Inventory (NPI; Bradwell et al [52], Chen et al [18], and Valentí-Soler et al [33]), the Behavioral Activity Rating Scale (BARS; Jøranson et al [28]), the Cohen-Mansfield Agitation Inventory–Short Form (CMAI-SF; Moyle et al [24]), and the Rating Anxiety in Dementia (RAID) scale (Petersen et al [34]). Mervin et al [27] was reclassified to narrative-only synthesis owing to cohort overlap with Moyle et al [24], and Robinson et al [44] was reclassified to narrative-only owing to its qualitative-observational design. The HKSJ pooled estimate was Hedges *g*=0.44 (95% CI 0.03-0.84; 95% PI −0.42 to 1.30; *I*²=67%; τ²=0.088; *P*=.04), indicating a small-to-moderate, statistically significant reduction in NPS favoring care robot intervention. Bradwell et al [52] reported the largest individual effect (Hedges *g*=1.05, 95% CI 0.52-1.59) using the Joy for All robot pet over a 4-month cluster RCT in care homes, with NPI subdomain reductions in delusions, depression, anxiety, elation, and apathy (all *P*≤.03). Valentí-Soler et al [33] demonstrated a sustained intervention effect at follow-up (BARS effect estimate 5.5, 95% CI 0.1-11.0; *P*=.048). Chen et al [18] reported no significant between-group difference on the CMAI-SF (adjusted mean difference −1.89, 95% CI −5.81 to 2.02; *P*=.34), and Valentí-Soler et al [33] showed null effects on total NPI score in their nursing home arm.

#### Quality of Life

Five studies [18,20,28,33,42] (n=472) contributed using the Quality of Life in Late-Stage Dementia scale, Quality of Life in Alzheimer’s Disease scale, Short Form 36 Emotional Wellbeing subscale, and the Frail Questionnaire [20]. The HKSJ pooled estimate was Hedges *g*=0.15 (95% CI −0.41 to 0.71; 95% PI −0.86 to 1.16; *I*²=65%; τ²=0.092; *P*=.51), indicating a small effect direction favoring care robots that did not reach statistical significance. Effect estimates were heterogeneous: Papadopoulos et al [42] reported a substantial benefit on Short Form 36 Emotional Wellbeing scale (Hedges *g*=1.14, 95% CI 0.23-2.04; *P*=.02; ηp²=.258), whereas Pollak et al [20] (n=220) detected no significant change in social or physical frailty (*P*=.42 and .99, respectively), and Valentí-Soler et al [33] observed a counter-direction effect on Quality of Life in Late-Stage Dementia scale in the day-care PARO arm (Hedges *g*=−0.27; *P*=.04 favoring control). Jøranson et al [29] reported QoL effects only in residents with severe dementia (overall *P*=.12, severe-subgroup *P*=.008), suggesting that effects may be conditional on dementia severity.

#### Depression

Seven studies (n=620) contributed using the Geriatric Depression Scale (GDS or GDS-SF [Geriatric Depression Scale – Short Form]) [18,20], the Cornell Scale for Depression in Dementia (CSDD [28,34]), the Strengths and Difficulties Questionnaire emotional subscale (SDQ [42]), the Child Behavior Checklist Internalizing subscale [36], and the NPI affective subdomain [33]. The HKSJ pooled estimate was Hedges *g*=0.20 (95% CI −0.08 to 0.49; 95% PI −0.36 to 0.76; *I*²=44%; τ²=0.039; *P*=.13), indicating a small effect direction favoring care robots that did not reach statistical significance. Jøranson et al [28] demonstrated a significant reduction at follow-up (CSDD effect estimate 3.9, 95% CI 0.4-7.3; *P*=.03); Petersen et al [34] reported a significant CSDD between-group difference (*P*=.001), though the directional interpretation of CSDD change scores in the published table is internally inconsistent with the abstract narrative (sensitivity analysis below). Contributions from Pollak et al [20] (GDS-SF *P*=.90), Chen et al [18] (GDS *P*=.33), and Papadopoulou et al [43] (Strengths and Difficulties Questionnaire emotional *P*=.80) drew the pooled estimate toward null.

#### Agitation

Four studies (n=484), all using PARO, contributed measurements via the BARS [28], CMAI-SF [24], RAID [34], and NPI agitation subdomain [33]. After excluding Mervin et al [27] for cohort overlap with Moyle et al [24], the HKSJ pooled estimate was Hedges *g*=0.32 (95% CI −0.07 to 0.71; 95% PI −0.26 to 0.89; *I*²=29%; τ²=0.018; *P*=.08), indicating a small-to-moderate effect direction favoring care robots that approached but did not reach conventional statistical significance. The pool was dominated by 2 large studies with discordant findings: Moyle et al [24] (n=271 in PARO vs Usual Care contrast) reported no significant CMAI-SF difference (adjusted mean difference −1.89, 95% CI −5.81 to 2.02; *P*=.34), while Petersen et al [34] (n=61) reported a significant between-group RAID difference (*P*=.003). Jøranson et al [28] demonstrated a sustained BARS reduction at follow-up (effect estimate 5.5, *P*=.048). The lower heterogeneity (*I*²=29%) reflects the removal of the overlapping cohort data from Mervin et al [27].

#### Stress and Pain

Six studies (n=504) contributed across markedly heterogeneous clinical contexts: procedural pain in children [22,45], chronic psychological distress in older adults [20,34], socially induced stress in healthy children [38], and social-affective regulation in children with autism [47]. The HKSJ pooled estimate was Hedges *g*=0.53 (95% CI −0.48 to 1.53; 95% PI −1.57 to 2.62; *I*²=90%; τ²=0.510; *P*=.24), reflecting extreme between-study heterogeneity (90%) and a wide PI encompassing both meaningful benefit and meaningful harm. Rossi et al [45] produced the largest individual effect (salivary cortisol decrease in NAO group, Hedges *g*=2.36; 95% CI 1.62-3.11; *P*<.001); Ali et al [22] showed a moderate benefit on Observational Scale of Behavioral Distress–Revised during intravenous insertion (Hedges *g*=0.37; *P*=.047); and Petersen et al [34] reported physiologic stress markers favoring PARO (galvanic skin variability, *P*<.001). However, Kitt et al [38] reported a directionally negative finding using the Positive and Negative Affect Schedule for Children – Short Form positive affect subscale (robot group showed greater decrease poststressor than control, Hedges *g*=−0.52; *P*=.04), and Pollak et al [20] detected no group difference (*P*=.99). Given the *I*² of 90%, the pooled estimate should be interpreted with caution; the qualitative pattern suggests benefits may be context-dependent (procedural and physiologic stress more responsive than self-reported affect).

#### Social-Communicative Skills

Five studies (n=151), with all 5 enrolling children with ASD and using humanoid platforms (NAO [19,36,46], CommU [39], and iRobiQ [50]), contributed via the Social Responsiveness Scale [19], Childhood Autism Rating Scale-2 [36], joint attention frequency [39], gesture production [46], and eye contact frequency [50]. The HKSJ pooled estimate was Hedges *g*=0.45 (95% CI −0.52 to 1.42; 95% PI −1.66 to 2.56; *I*²=75%; τ²=0.453; *P*=.27), indicating a moderate effect direction favoring care robots that did not reach statistical significance under HKSJ adjustment. Three individual studies achieved significance: Kumazaki et al [39] (joint attention frequency, Hedges *g*=1.07; *P*=.01), So et al [46] (gesture production, Hedges *g*=1.07; *P*=.01), and De Korte et al [19] for the Functional self-initiations subtype (overall Hedges *g*=0.52; *P*=.05 approximately; functional subtype total self-initiations, time×group estimate −0.19, 95% CI −0.38 to 0.02; functional self-initiations, time×group estimate −0.27, 95% CI −0.50 to −0.04). However, Holeva et al [36] reported no statistically significant between-group difference on Childhood Autism Rating Scale-2 Total (group-by-time interaction *P*=.16), with both groups improving similarly within-subject. Yun et al [50] reported similar improvements in eye contact across robot and therapist groups (*P*=.50). The wide HKSJ-adjusted CI is partly driven by the small overall sample (n=151) and the conservative HKSJ small-sample correction.

#### Cognitive Function

Four studies (n=461) contributed via the Mini-Mental State Examination (MMSE) [33], the Montreal Cognitive Assessment (MoCA) [18], the Short Portable Mental Status Questionnaire [20], and the CATS Test of visuospatial hemineglect in poststroke patients [21]. The HKSJ pooled estimate was Hedges *g*=0.18 (95% CI −0.62 to 0.98; 95% PI −1.11 to 1.47; *I*²=71%; τ²=0.102; *P*=.52), reflecting a null-centered direction with substantial heterogeneity. Karner et al [21] showed a large effect on hemineglect improvement (Hedges *g*=1.13, 95% CI 0.46-1.81; *P*=.001), but this measure assesses a narrow poststroke cognitive domain rather than general cognitive function in dementia. The remaining 3 studies in older adults with dementia detected no significant between-group differences on global cognition measures (Chen et al [18] MoCA *P*=.07; Valentí-Soler et al [33] MMSE *P*=.28; Pollak et al [20] Short Portable Mental Status Questionnaire *P*=.69). Heterogeneity therefore reflects domain divergence between hemineglect rehabilitation and general cognitive screening rather than within-domain variability.

### Subgroup and Sensitivity Analyses

#### Sensitivity Analysis: Data Extraction Ambiguity

During full-text data extraction, we identified an internal inconsistency in Petersen et al [34] regarding the directional interpretation of RAID and CSDD change scores. Table 2 in Petersen et al [34] reports “difference (post–pre)” with positive values for the treatment group (RAID 2.5, SE 0.6; CSDD 2.81, SE 0.4) and small positive values for controls (RAID 0.55, SE 0.2; CSDD 0.78, SE 0.4); the abstract states scores “were increased in the treatment group” while the conclusion describes “decreased stress and anxiety.” We interpreted these change values as the magnitude of symptom decrease and conducted a sensitivity analysis excluding Petersen et al [34] from each pool to which it contributed. When Petersen et al [34] was excluded, NPS pool ceased to reach statistical significance (Hedges *g*=0.40, 95% CI −0.11 to 0.91; *P*=.095; k=5); depression remained near null (Hedges *g*=0.08, 95% CI −0.14 to 0.30; k=6); agitation pool point estimate shifted slightly (Hedges *g*=0.23, 95% CI −0.20 to 0.65; k=3); stress and pain remained nonsignificant (Hedges *g*=0.48, 95% CI −0.84 to 1.81; k=5). We retain Petersen et al [34] in the main analyses while flagging this as a data extraction limitation; importantly, no statistically significant pooled benefit is fully robust to Petersen exclusion, underscoring the modest evidentiary base for the field overall.

#### Sensitivity Analysis: LOO and Data Quality

We further conducted LOO sensitivity analyses for the only outcome reaching statistical significance (NPS pool, k=6). Under LOO, the pooled effect retained statistical significance in only 1 of 6 iterations (excluding Valenti-Soler 2015 [33]: Hedges *g*=0.54, 95% CI 0.11-0.97; *P*=.03); in the remaining 5 iterations, the 95% CI crossed null (excluding Bradwell et al [52]: *P*=.07; Chen et al [18]: *P*=.09; Jøranson et al [28]: *P*=.09; Moyle et al [24]: *P*=.051; and Petersen et al [34]: *P*=.095). Restricting to studies whose mean and SD values were extracted directly from published tables (class A: Bradwell et al [52], Chen et al [18], Jøranson et al [28]; k=3) yielded Hedges *g*=0.67 (95% CI −0.10 to 1.43; *P*=.07), losing statistical significance owing to the small k under HKSJ small-sample correction. These LOO results demonstrate that the single statistically significant pooled effect in our analysis is fragile, as it depends on the joint contribution of all 6 [18,24,29,33,34,52] contributing studies and is sensitive to the exclusion of any individual study. We therefore interpret the NPS result as suggestive of benefit rather than as definitive evidence, and emphasize that adequately powered, multicenter confirmatory trials are required.

In robot-type subgroup analyses, PARO was associated with the most consistent single-study effects on NPS among older adults with dementia (notably Jøranson et al [28] BARS *P*=.048; Petersen et al [34] RAID *P*=.003 with the data ambiguity flag noted above), although the small per-arm subgroup k (=4) limits formal subgroup pooling. Humanoid platforms (NAO, Kaspar, CommU, and iRobiQ) supplied all 5 [19,36,39,46,50] contributing studies in the social-communicative skills pool. Ad hoc duration-based subgroup analysis suggested numerically larger effects in studies with intervention durations ≥10 weeks (eg, Bradwell et al [52] 16 weeks, Jøranson et al [28] 12 weeks, and Petersen et al [34] 12 weeks) compared with shorter pilots, but the small subgroup k precludes formal heterogeneity testing across this dimension. Sensitivity analyses excluding the 4 [18,20,21,33] studies rated overall high RoB 2 risk of bias did not materially alter pooled point estimates (within ±0.06 Hedges g of primary analyses) and did not reverse any nonsignificant pool to significance.

### Reporting Biases and Small-Study Effects

**Figure 4.**
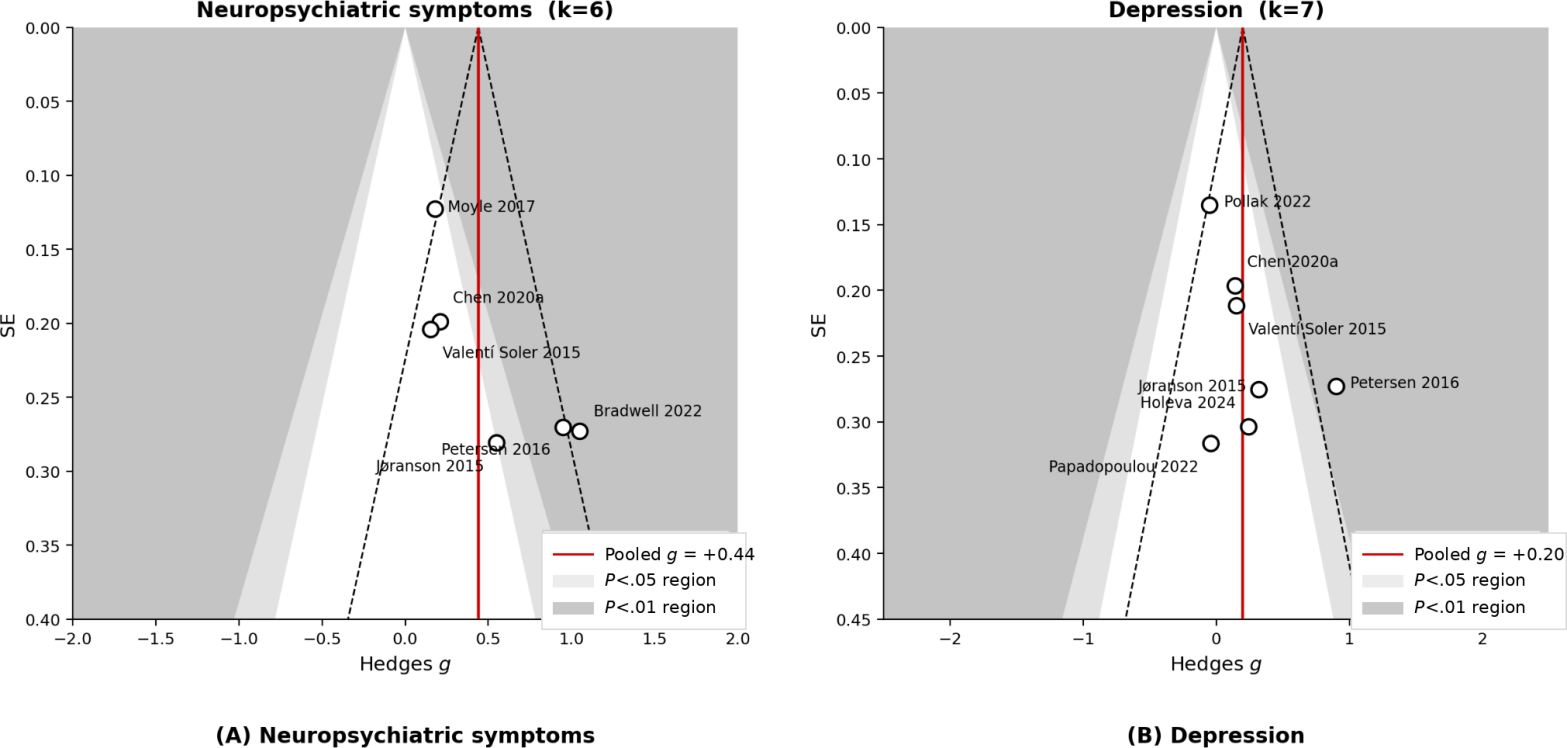
Contour-enhanced funnel plots for the 2 domains with the most contributing trials: (A) neuropsychiatric symptoms (k=6) and (B) depression (k=7). The vertical red dashed line marks the Hartung-Knapp-Sidik-Jonkman pooled estimate; diagonal dashed lines show pseudo 95% confidence limits. Because no domain reached k≥10, asymmetry was not formally tested; plots are shown for visual transparency only [18,20,24,29,33,34,36,43,52].

Contour-enhanced funnel plots for NPS (k=6) and depression (k=7) are shown in Figure 4. Both domains have k<10, below the recommended threshold for formal Egger regression test [60]; accordingly, funnel plots are reported for visual transparency only. Visual inspection of the NPS funnel plot showed scattering largely consistent with the pooled estimate; the depression funnel plot appeared broadly symmetric. We did not formally test funnel plot asymmetry given the small k. Sources of small-study effects could include publication bias, methodological differences, true between-study effect heterogeneity, or chance—none of which can be reliably distinguished with k<10.

### Certainty of Evidence

Full GRADE ratings are presented in Table 2. In summary, certainty was rated low for one outcome—NPS—reflecting structural performance bias inherent to unblinded robot trials and substantial inconsistency, partially offset by the directional consistency of effect across most contributing studies. Certainty was rated very low for the remaining 6 outcomes—quality of life, depression, agitation, stress and pain, social-communicative skills, and cognitive function—reflecting a combination of nonsignificant or null pooled estimates, imprecision (95% CIs crossing null and wide PIs relative to effect size), and small contributing sample sizes. The Discussion section is framed in light of these certainty ratings—that is, the single statistically significant pooled effect (NPS, sensitive to one study’s inclusion) is interpreted as a suggestive signal supporting careful, selective consideration of care robots in narrow indications, rather than as confirmatory evidence supporting general clinical recommendations or health system integration.

### Acceptance and Implementation Outcomes (Secondary)

A minority of included studies reported acceptance- or usability-relevant secondary outcomes (eg, System Usability Scale, Unified Theory of Acceptance and Use of Technology items, observed session completion rates, and facilitator-time data). Because reporting was sparse, inconsistent, and predominantly descriptive, these outcomes were summarized narratively rather than pooled. Across the studies that reported them, acceptance was generally higher in sessions with dedicated facilitator support and in longer-duration protocols.

## Discussion

### Principal Findings

Relative to the 4 prespecified objectives, this synthesis produced a patterned rather than uniform set of findings. Across the 7 prespecified outcome domains, HKSJ random-effects meta-analysis identified a single domain—NPS—for which the pooled estimate favored care robots and the confidence interval excluded the null. For the remaining 6 domains (quality of life, depression, agitation, stress and pain, social-communicative skills, and cognitive function), the pooled estimates were directionally favorable but did not reach statistical significance. Critically, a distinction must be drawn between the average effect and the distribution of effects across settings; whereas the CI reflects uncertainty about the mean effect, the PI describes the plausible effect in a new setting. In every domain—including NPS—the 95% PI encompassed the null, so although an average benefit is detectable across existing trials for NPS, a beneficial effect cannot be assumed in a new care setting. The neuropsychiatric-symptom signal was, moreover, fragile, losing significance under most LOO exclusions and upon removal of the single study with documented data extraction ambiguity. Interpreted strictly in light of the substantial between-study heterogeneity observed across pools, the predominance of high or some-concerns RoB 2 ratings, and GRADE certainty ranging from low (NPS) to very low (all other domains), these results indicate that no pooled estimate currently provides a robust basis for clinical decision-making.

### Interpretation in the Context of Prior Reviews

Our findings both converge with and extend prior syntheses. For older adults with dementia, the direction and approximate magnitude of the pooled effects we observed for NPS and agitation are consistent with the single-population meta-analyses of Pu et al [11], Leng et al [12], and Yu et al [13], none of which, however, compared these effects with those observed in pediatric populations. For social-communicative skills in children with ASD, our humanoid-platform effect size is directionally consistent with Scassellati et al [6] but, under HKSJ pooling, did not reach statistical significance (Hedges *g*=0.45, 95% CI −0.52 to 1.42). The principal methodological contribution of this review is the direct juxtaposition of platform–population pairings within a single analytic framework. The pattern of variation across robot platforms and populations observed here is therefore best read as a hypothesis-generating descriptive signal rather than as confirmatory evidence; it nonetheless points to platform-population matching, rather than platform-agnostic deployment, as a useful organizing principle for future trial design and procurement decisions.

Our nonsignificant pooled effects on depression and cognitive function are informative rather than merely negative. Depression in dementia is etiologically complex, with substantial neurobiological determinants that brief psychosocial interventions may be insufficient to modify [62]. The borderline signal in our exploratory subgroup restricted to PARO interventions of ≥10 weeks is hypothesis-generating only and requires prospective adequately powered testing. The null pooled effect for cognitive function is consistent with the broader behavioral-intervention literature and reflects the well-established limitation of noncognitive behavioral inputs in reversing underlying neurodegeneration [40,62-65]; measurement-level sensitivity of global screening instruments (MMSE and MoCA) to detect short-term changes may also contribute.

### Care Setting as a Moderator

Setting was not a prespecified subgroup analysis, but the observed effect pattern suggests it warrants interpretation. The domains with the largest pooled effects—NPS and agitation (dementia in long-term care) and social-communicative skills (ASD therapy, predominantly in structured therapy or hospital outpatient settings)—are those in which the intervention was delivered in a structured daily routine with consistent facilitator support. Community and home-setting deployments were underrepresented (n=2) and reported smaller and less consistent effects. The available evidence therefore best supports deployment in settings where the surrounding care structure—scheduled sessions, trained facilitators, documentation routines—can be aligned with the intervention [66], and does not yet speak to unstructured community or home deployment.

### Targeted Adjunctive Use Cases

Rather than supporting broad claims about health-system integration, the evidence pattern above—interpreted in the context of low GRADE certainty for the one statistically significant outcome (NPS, sensitive to a single study’s inclusion) and very low certainty for the remaining 6 nonsignificant outcomes—is most consistent with 3 narrow adjunctive use cases in which the available evidence and the clinical realities align. These should be regarded as candidate indications meriting further confirmatory trials, not as recommendations for routine clinical adoption.

1. Facilitator-supported calming and engagement sessions for older adults with moderate-to-severe dementia in long-term care. PARO-based sessions of approximately 20‐30 minutes, delivered 2‐3 times per week by trained staff for ≥10 weeks, are the intervention parameters most consistently associated with detectable effects on NPS (agitation pooled effect was directionally similar but did not reach conventional statistical significance). GRADE certainty for this configuration is low. The role is adjunctive—complementing, not replacing, person-centered nursing care—and is most appropriate for units where baseline neuropsychiatric-symptom burden is high and nonpharmacological options are prioritized.
2. Procedural distraction in pediatric inpatient settings. Humanoid robots deployed during time-limited, anxiety-provoking procedures (eg, venipuncture, imaging, and preoperative induction) represent a plausible adjunctive use supported by the stress, pain, and engagement literature [67,68], though with very low GRADE certainty. The time-limited nature of the exposure is itself a strength here: novelty effects, which are a limitation in longer-horizon claims, may be an asset in single-session procedural use.
3. Structured social-communicative practice tasks for children with ASD. Humanoid robot–assisted sessions are best positioned as a structured-practice adjunct to established behavioral therapies such as applied behavior analysis or pivotal response treatment, not as a stand-alone therapeutic modality. GRADE certainty is very low (the social-communicative skills pool did not reach statistical significance under HKSJ pooling), and transfer of gains from robot-mediated to human-mediated social interaction remains an unresolved question that future trials should address [69,70]

### Clinical Effectiveness, Technology Acceptance, and Implementation Feasibility

The 3 dimensions of clinical effectiveness (does the intervention work under trial conditions?), technology acceptance (will patients, families, and staff use it?), and implementation feasibility (can the intervention be embedded sustainably in routine care?) are related but distinct, and the evidence base for each differs in maturity. This review quantifies clinical effectiveness and finds a patterned but uneven picture. Technology acceptance is supported by a parallel body of qualitative and survey-based literature [7,8,71] indicating generally positive acceptance when sessions are facilitator-supported, culturally aligned, and adequately introduced; but acceptance ratings are variable across populations and platforms, and this review did not pool these data. Implementation feasibility remains the least-developed dimension: we did not identify studies that reported cost-effectiveness, institutional readiness, or sustained-deployment outcomes with the rigor required for pooled synthesis. Conflating these 3 dimensions risks overstating the readiness of care-robot deployment; they should be reported separately in future trials and reviews.

Acceptability is decisive for translation yet was not poolable in this effectiveness-focused synthesis, and it is especially salient in older adults, in whom acceptance is shaped by prior attitudes, perceived usefulness, and design rather than being a given. Dedicated work in this population—including the development of instruments to measure acceptance of social robotics and willingness-to-interact studies—underscores that acceptance is a distinct, measurable construct that should be assessed in its own right rather than inferred from efficacy [72,73].

### Clinical and Implementation Implications

From a digital health and nursing informatics perspective, these findings, interpreted in light of low or very low GRADE certainty across all 7 outcome domains, suggest several conditional implementation implications rather than firm recommendations. First, where care robots are considered for selective adoption in the narrow indications identified above, they would be most appropriately positioned within the broader digital health ecosystem rather than deployed as isolated devices. PARO and humanoid robots equipped with sensor arrays and data-logging capabilities can generate structured patient engagement metrics—interaction duration, motion activity, physiological indicators—that may be interoperable with electronic health records and clinical decision-support systems where local infrastructure permits. The clinical value of such integration has not been formally evaluated in the trials reviewed here [74].

### Strengths and Limitations

Strengths of this review include a cross-population, cross-platform quantitative synthesis; use of the HKSJ random-effects estimator with PIs rather than CIs alone; explicit distinction between average effects and their distribution across settings; PRISMA 2020, PRISMA 2020 for Abstracts, and PRISMA-S adherence with all checklists supplied; dual-reviewer screening, extraction, risk-of-bias assessment, and GRADE rating throughout; and a comprehensive search of 5 databases and 2 trial registries without language or date restrictions.

Limitations should be read in light of the cautious claims above. First, the review was not prospectively registered in PROSPERO. Although an a priori protocol was developed and is available on request, the absence of public prospective registration reduces independent verifiability of prespecification claims. Second, performance bias is structurally inherent to care robot research: blinding of participants and care providers to robotic versus nonrobotic interaction is impossible, and this domain consistently represented the highest RoB 2 ratings, rated high or some concerns in all 34 [18-22,24-52] included studies. Attention-matched active control conditions (Moyle et al [24] plush and Yun et al [50] therapist-guided routine) partially mitigate this concern but cannot eliminate it. Third, considerable clinical and methodological heterogeneity remains across pools, ranging from low to high, reflecting diversity in robot platforms, target populations, intervention dose, and outcome instruments. Fourth, the included sample remains modest, with a median per-study analyzed n=62 and only 4 [20,24,25,27] included studies enrolling >200 participants. Fifth, follow-up durations were generally short, with most studies measuring outcomes at end-of-intervention (≤16 weeks); long-term sustainability and dose-response relationships remain underexplored. Sixth—and new in this revision—primary-source data verification revealed limitations specific to data extraction: (1) one cohort overlap (Mervin et al [27] with Moyle et al [24] cluster RCT, n=415 Australian residents) was identified during full-text reextraction and resolved via reclassification of Mervin et al [27] to narrative-only synthesis under Senn [58]; (2) Robinson et al [44] was confirmed as a qualitative observational follow-up of an external RCT cohort and reclassified to narrative-only; and (3) Petersen et al [34] published a table whose directional interpretation of RAID and CSDD change scores is inconsistent with the abstract narrative, prompting a sensitivity analysis. When Petersen was excluded, the only statistically significant pool (NPS) became nonsignificant under HKSJ pooling, highlighting the modest evidentiary base. Finally, 3 included studies were small pilot RCTs (Marino et al [41] n=14; Yun et al [50] n=15; Srinivasan et al [47] n=24), and several studies analyzed secondary outcomes rather than preregistered primary endpoints. Together, these limitations argue for caution in interpreting any single pooled estimate and for prioritizing future adequately powered, multicenter RCTs with preregistered primary outcomes. A further limitation specific to data extraction quality is that mean and SD values for 9 of 37 study-outcome contributions (24%) were derived from text or figure approximations rather than from published tables; sensitivity analyses excluding these contributions did not change whether any pool reached statistical significance, but produced moderate changes in pooled point estimates (notably a substantial attenuation of the stress and pain estimate when text or figure-approximated contributions were excluded). To make the link to certainty explicit, these limitations map directly onto the GRADE downgrade domains recorded in Table 2. The structural impossibility of blinding and the predominance of high or some concerns RoB 2 ratings drive the risk-of-bias downgrade; the wide range of *I*² across pools drives the inconsistency downgrade; and the modest per-study sample sizes, together with CIs crossing the null and wide PIs, drive the imprecision downgrade. Reliance on text- or figure-approximated data for a subset of contributions bears on both the risk of bias and imprecision judgments. The deliberate cross-population pooling, although a strength for breadth, introduces a degree of indirectness when pooled estimates are applied to any single target population; we flag this as a consideration even though it did not by itself trigger a separate GRADE downgrade in our profile.

### Future Research Directions

Several priorities emerge directly from the gaps identified above. Multicenter RCTs of adequate size, with attention-matched active controls and assessor-blinded outcome measurement, are the most immediate methodological need; sample sizes should be informed by the HKSJ-corrected effect estimates reported here, which are typically more conservative than earlier DerSimonian-Laird–based values and therefore produce larger sample size targets. Standardization of outcome measurement through a stakeholder-derived core outcome set, developed along lines analogous to the COMET Initiative [8], would substantially improve comparability across future trials. Follow-up periods of 3, 6, and 12 months postintervention are necessary to distinguish short-term engagement from durable clinical benefit—a distinction that current short-duration evidence cannot address. Formal cost-effectiveness analyses and budget-impact modeling are required to inform health technology assessment. Implementation science studies that explicitly measure staff training requirements, institutional readiness, and patient and family acceptance as implementation outcomes in their own right would begin to build the implementation-feasibility evidence base that is currently absent. Cross-cultural replication, particularly in low- and middle-income settings, and mechanistic studies pairing behavioral outcomes with physiological markers (cortisol and heart rate variability) or neuroimaging where feasible, round out the research agenda.

### Conclusions

This systematic review and meta-analysis provides one of the broadest quantitative syntheses to date of care-robot RCTs across patient populations and outcome domains, and is best read as an exploratory, hypothesis-generating synthesis rather than a definitive one. Following systematic full-text data verification of all included randomized controlled studies, a statistically significant pooled effect favoring care robots emerged in only one of the 7 prespecified domains, NPS. That signal proved fragile under LOO and data extraction sensitivity analyses, and its PI, like those of the remaining 6 domains, encompassed the null, so a benefit cannot be assumed in any new care setting. Across the other 6 domains, pooled effects favored care robots in direction but did not reach statistical significance. Interpreted strictly in light of substantial between-study heterogeneity, the predominance of high or some concerns risk-of-bias ratings, and GRADE certainty of low (NPS) to very low (all other domains), these findings indicate that the evidence base is more limited than earlier syntheses suggested, a difference attributable to the HKSJ small-sample correction, primary-source data verification, and PI reporting. The current evidence is therefore promising but not yet sufficient to support routine clinical adoption of care robots across the breadth of populations and outcomes examined. Where benefits may be most plausibly realized, neuropsychiatric symptom reduction in older adults with dementia and procedural-distress mitigation in pediatric settings, they should be regarded as candidate indications requiring confirmation, not as established uses. Accordingly, care robots are best positioned as facilitator-supported adjuncts that augment, rather than substitute for, human-delivered care, and broader implementation claims should await adequately powered, multicenter trials with attention-matched controls, blinded outcome assessment, and extended postintervention follow-up.

## Supplementary material

10.2196/95232 Multimedia Appendix 1 Characteristics of the 34 included randomized and cluster-randomized controlled trials of care robot interventions (per-study; full version), conducted across 17 countries and published 2015-2024. Target populations include older adults with dementia or mild cognitive impairment, children and adolescents with autism spectrum disorder, hospitalized or procedurally distressed pediatric patients, and other adult clinical populations. Robot platforms include PARO, humanoid platforms (NAO, Pepper, Kabochan, CommU, and Kaspar), and other companion and pet platforms. Cohort-overlap allocation per Senn (2009) is documented in the footnote.

10.2196/95232 Checklist 1 PRISMA-S checklist.

10.2196/95232 Checklist 2 PRISMA 2020 for Abstracts checklist.

10.2196/95232 Checklist 3 PRISMA Expanded checklist.

## Abbreviations

ASD: autism spectrum disorder
BARS: Behavioral Activity Rating Scale
CMAI-SF: Cohen-Mansfield Agitation Inventory–Short Form
CSDD: Cornell Scale for Depression in Dementia
GDS: Geriatric Depression Scale
GDS-SF: Geriatric Depression Scale – Short Form
GRADE: Grading of Recommendations Assessment, Development and Evaluation
HKSJ: Hartung-Knapp-Sidik-Jonkman
ICTRP: International Clinical Trials Registry Platform
LOO: leave-one-out
MeSH: Medical Subject Headings
MMSE: Mini-Mental State Examination
MoCA: Montreal Cognitive Assessment
NPI: Neuropsychiatric Inventory
NPS: neuropsychiatric symptoms
PI: prediction interval
PRISMA: Preferred Reporting Items for Systematic Reviews and Meta-Analyses
PRISMA-S: Preferred Reporting Items for Systematic Reviews and Meta-Analyses literature search
PROSPERO: International Prospective Register of Systematic Reviews
RAID: Rating Anxiety in Dementia
RCT: randomized controlled trial
ROB 2: Risk of Bias 2
WHO: World Health Organization
