# Supplementary material for: Care Robots as Emerging Health Technologies: Systematic Review and Meta-Analysis of Randomized Controlled Trials
Source: J Med Internet Res. 2026 Jun 30;28:e95232. doi: 10.2196/95232 (PMC13318203; doi:10.2196/95232)
Supplement: Multimedia Appendix 1 [file jmir-v28-e95232-s001.docx]

**Table S1.** Characteristics of the 34 included randomized and cluster-randomized controlled trials of care robot interventions (per-study; full version), conducted across 17 countries and published 2015–2024. Target populations include older adults with dementia or mild cognitive impairment, children and adolescents with autism spectrum disorder, hospitalized or procedurally distressed pediatric patients, and other adult clinical populations. Robot platforms include PARO, humanoid platforms (NAO, Pepper, Kabochan, CommU, Kaspar), and other companion/pet platforms. Cohort-overlap allocation per Senn (2009) is documented in the footnote.

| **No.[Ref]** | **Study (Year)** | **Country** | **Design** | **Population (Age Range)** | **n Rand. (Analyzed)** | **Robot Type / Model** | **Control Condition** | **Duration** | **Primary Outcome Measures** |
| --- | --- | --- | --- | --- | --- | --- | --- | --- | --- |
| 1 [22] | Ali et al. (2021) | Canada | RCT | Children undergoing IV insertion (6-11 y) | 86 (81) | Humanoid (unnamed) | Standard care | Single session | Behavioral distress (OSBD-R); pain (FPS-R) |
| 2 [51] | Boumans et al. (2020) | Netherlands | RCT | Outpatients, elderly (60-91 y) | 78 (75) | Social robot (unnamed) | Non-robot care | Single session | Consumer Quality Index |
| 3 [52] | Bradwell et al. (2022) | UK | Cluster RCT | Dementia, care home residents | 83 (63) | Joy for All (pet robot) | Usual care | 4 months | Neuropsychiatric Inventory (NPI, 12 subdomains) |
| 4 [18] | Chen et al. (2020a) | Hong Kong | Cluster RCT | Dementia, LTC residents (67-108 y) | 103 | Kabochan | Usual care | 32 weeks (ABA) | NPI-Q; GDS; MoCA; QoL-AD; ADL |
| 5 [31] | Chen et al. (2020b) | Hong Kong | Cluster RCT | Dementia, LTC residents (67-108 y) | 103 | Kabochan | Usual care | 32 weeks | Technology acceptance (STAM) |
| 6 [19] | De Korte et al. (2020) | Netherlands | RCT | ASD, children (3-8 y) | ~44 (32) | NAO | Standard PRT | 20 sessions | Self-initiations; SRS (parent/teacher report) |
| 7 [35] | Feingold-Polak et al. (2021) | Israel | RCT | Post-stroke rehabilitation (30-77 y) | 24 (18) | Pepper | Computer-based rehab | 5-7 weeks | USEQ satisfaction score |
| 8 [36] | Holeva et al. (2024) | Greece | RCT | ASD, children (6-12 y) | 51 (44) | NAO | Therapist only | 12 weeks | CARS-2; NEPSY-2; CBCL; SDQ |
| 9 [37] | Johanson et al. (2019) | New Zealand | Parallel RCT | Young adults (>16 y) | 181 (174) | NAO (receptionist) | 4 conditions (no robot) | Single session | Engagement; empathy; attention |
| 10 [26] | Jones et al. (2018) | Australia | Cluster RCT | Dementia (>60 y) | 415 | PARO | Reading / usual care | 10 weeks | Engagement; mood (video coding) |
| 11 [28] | Joranson et al. (2015) | Norway | Cluster RCT | Dementia, nursing home (62-95 y) | 60 (53) | PARO | Usual care | 12 weeks | BARS agitation; CSDD depression |
| 12 [29] | Joranson et al. (2016) | Norway | Cluster RCT | Dementia, nursing home (62-95 y) | 60 (53) | PARO | Usual care | 12 weeks | QUALID quality of life |
| 13 [30] | Joranson et al. (2021) | Norway | Cluster RCT | Dementia, nursing home (62-95 y) | 60 (39) | PARO | Usual care | 12 weeks | Sleep quality (actigraphy) |
| 14 [21] | Karner et al. (2019) | Austria | RCT | Post-stroke, elderly (>60 y) | 47 (39) | PARO | Read-aloud activity | 2 weeks | SINGER (self-care, mobility, cognition) |
| 15 [38] | Kitt et al. (2021) | USA | RCT | Children (7-10 y), stress paradigm | 70 | SAR (MiRo) | Robot-absent condition | TSST-C session | PANAS-C-S; SAM (perceived stress) |
| 16 [39] | Kumazaki et al. (2018) | Japan | RCT | ASD + typically developing children (5-6 y) | 68 | CommU | Human conversational partner | 3 sessions | Joint attention frequency |
| 17 [40] | Logan et al. (2019) | USA | RCT | Hospitalized children (3-10 y) | ~80 | Interactive SR bear | Tablet / plush toy | Single session | Anxiety; affect; pain |
| 18 [41] | Marino et al. (2020) | Italy | RCT | ASD, children (4-8 y) | 14 | NAO | CBT without robot | 16 sessions | Emotion comprehension (TEC; ELT) |
| 19 [27] | Mervin et al. (2018) | Australia | Cluster RCT | Dementia, LTC (>60 y) | 209 | PARO | Plush toy / usual care | 10 weeks | CMAI-SF agitation; medication cost |
| 20 [24] | Moyle et al. (2017) | Australia | Cluster RCT | Dementia, LTC (>60 y) | 415 | PARO | Plush toy / usual care | 15 weeks | Engagement (video coding); CMAI-SF |
| 21 [25] | Moyle et al. (2018) | Australia | Cluster RCT | Dementia, LTC (>60 y) | 415 (175) | PARO | Plush toy / usual care | 10 weeks | Motor activity; sleep (actigraphy) |
| 22 [42] | Papadopoulos et al. (2021) | UK | Single-blind RCT | Care home residents (>65 y) | 33 (22) | Pepper (CARESSES) | Limited AI / usual care | 2 weeks | SF-36 (QoL); loneliness scale |
| 23 [43] | Papadopoulou et al. (2022) | Greece | RCT | SpLD, children (8-9 y) | 40 | NAO | Special educator only | 3 months | Language proficiency; SDQ; CBCL |
| 24 [34] | Petersen et al. (2016) | USA | Block design RCT | Dementia, LTC residents | 61 | PARO | Standard activity | 3 months | RAID anxiety; CSDD; stress (GSR, HR) |
| 25 [20] | Pollak et al. (2022) | Israel | RCT | Community-dwelling elderly (>65 y) | 220 (175) | Robotic pet | Usual care | 30 days | Social/physical frailty; GDS-SF; SPMSQ |
| 26 [44] | Robinson & Broadbent (2016) | New Zealand | RCT | Elderly, aged-care facility | ~40 | PARO | Usual care | 12 weeks | NPI; mood; psychosocial benefits |
| 27 [45] | Rossi et al. (2022) | Italy | RCT | Hospitalized children (3-10 y) | 73 (48) | NAO | Nurse / parents | Single session | Emotion (TEC); stress; temperament (CBQ) |
| 28 [46] | So et al. (2018) | Hong Kong | RCT | ASD, children (~9 y) | ~30 | NAO | Waitlist control | 12 weeks | Gesture production |
| 29 [47] | Srinivasan et al. (2015) | USA | Pilot RCT | ASD, children (5-12 y) | 36 | NAO (rhythm/robot) | Standard care | 10 weeks | Repetitive behavior; affect states |
| 30 [48] | Stoevesandt et al. (2021) | Germany | RCT | MRI outpatients (18-90 y) | 117 | Humanoid (unnamed) | Tablet-based information | Single session | Acceptance; health knowledge |
| 31 [33] | Valenti-Soler et al. (2015) | Spain | RCT | Dementia, nursing home (58-101 y) | ~101 | PARO + NAO | Usual care / real dog | 3 months | MMSE; NPI; QUALID; APADEM-NH |
| 32 [32] | van den Berk-Smeekens et al. (2021) | Netherlands | RCT | ASD, children (3-8 y) | 81 | NAO | PRT / TAU | 20 sessions | SRS; CGI-I; ADOS-2 |
| 33 [49] | Wei et al. (2021) | Taiwan | RCT | Elderly (>50 y) | 60 | Robot tutor (unnamed) | Video lecture | 90 min x 4 | Health literacy; RIMMS; flow state |
| 34 [50] | Yun et al. (2017) | South Korea | RCT | ASD, children (4-7 y) | 15 | iRobiQ | Therapist-led session | 8 weeks | Eye contact frequency; facial emotion recognition |

ABA, alternating baseline/treatment; ADOS-2, Autism Diagnostic Observation Schedule (2nd ed.); ADL, Activities of Daily Living; ASD, autism spectrum disorder; BARS, Brief Agitation Rating Scale; CARS-2, Childhood Autism Rating Scale (2nd ed.); CMAI-SF, Cohen-Mansfield Agitation Inventory–Short Form; CSDD, Cornell Scale for Depression in Dementia; FPS-R, Faces Pain Scale–Revised; GDS, Geriatric Depression Scale; LTC, long-term care; MMSE, Mini-Mental State Examination; MoCA, Montreal Cognitive Assessment; NAO, humanoid robot (SoftBank Robotics); NPI(-Q), Neuropsychiatric Inventory (Questionnaire); OSBD-R, Observational Scale of Behavioral Distress–Revised; PARO, therapeutic robot seal; QUALID, Quality of Life in Late-Stage Dementia; QoL-AD, Quality of Life–Alzheimer Disease; RAID, Rating Anxiety in Dementia; RCT, randomized controlled trial; SAR, socially assistive robot; SDQ, Strengths and Difficulties Questionnaire; SF-36, Short Form Health Survey; SRS, Social Responsiveness Scale; TAU, treatment as usual. Cohort-overlap allocation per Senn (2009) is documented above (see Methods)

**References**

18. Chen K, Lou VWQ, Tan KCK, Wai MY, Chan LL. Effects of a humanoid companion robot on dementia symptoms and caregiver distress for residents in long-term care. J Am Med Dir Assoc. Nov 2020;21(11):1724-1728. [doi: 10.1016/j.jamda.2020.05.036] [Medline: 32713772]

19. De Korte MW, van den Berk-Smeekens I, van Dongen-Boomsma M, et al. Self-initiations in young children with autism during pivotal response treatment with and without robot assistance. Autism. Nov 2020;24(8):2117-2128. [doi: 10.1177/1362361320935006] [Medline: 32730096]

20. Pollak C, Wexler SS, Drury L. Effect of a robotic pet on social and physical frailty in community-dwelling older adults: a randomized controlled trial. Res Gerontol Nurs. 2022;15(5):229-237. [doi: 10.3928/19404921-20220830-01] [Medline: 36113009]

21. Karner S, Stenner H, Spate M, Behrens J, Krakow K. Effects of a robot intervention on visuospatial hemineglect in postacute stroke patients: a randomized controlled trial. Clin Rehabil. Dec 2019;33(12):1940-1948. [doi: 10.1177/0269215519865993] [Medline: 31409126]

22. Ali S, Manaloor R, Ma K, et al. A randomized trial of robot-based distraction to reduce children’s distress and pain during intravenous insertion in the emergency department. Can J Emerg Med. Jan 2021;23(1):85-93. [doi: 10.1007/s43678-020-00023-5] [Medline: 33683608]

24. Moyle W, Jones CJ, Murfield JE, et al. Use of a robotic seal as a therapeutic tool to improve dementia symptoms: a cluster-randomized controlled trial. J Am Med Dir Assoc. Sep 1, 2017;18(9):766-773. [doi: 10.1016/j.jamda.2017.03.018] [Medline: 28780395]

25. Moyle W, Jones C, Murfield J, et al. Effect of a robotic seal on the motor activity and sleep patterns of older people with dementia, as measured by wearable technology: a cluster-randomised controlled trial. Maturitas. Apr 2018;110:10-17. [doi: 10.1016/j.maturitas.2018.01.007] [Medline: 29563027]

26. Jones C, Moyle W, Murfield J, et al. Does cognitive impairment and agitation in dementia influence intervention effectiveness? Findings from a cluster-randomized-controlled trial with the therapeutic robot, PARO. J Am Med Dir Assoc. Jul 2018;19(7):623-626. [doi: 10.1016/j.jamda.2018.02.014] [Medline: 29656838]

27. Mervin MC, Moyle W, Jones C, et al. The cost-effectiveness of using PARO, a therapeutic robotic seal, to reduce agitation and medication use in dementia: findings from a cluster-randomized controlled trial. J Am Med Dir Assoc. Jul 2018;19(7):619-622. [doi: 10.1016/j.jamda.2017.10.008] [Medline: 29325922]

28. Jøranson N, Pedersen I, Rokstad AMM, Ihlebaek C. Change in quality of life in older people with dementia participating in Paro-activity: a cluster-randomized controlled trial. J Adv Nurs. Dec 2016;72(12):3020-3033. [doi: 10.1111/jan.13076] [Medline: 27434512]

29. Jøranson N, Pedersen I, Rokstad AMM, Ihlebæk C. Effects on symptoms of agitation and depression in persons with dementia participating in robot-assisted activity: a cluster-randomized controlled trial. J Am Med Dir Assoc. Oct 1, 2015;16(10):867-873. [doi: 10.1016/j.jamda.2015.05.002] [Medline: 26096582]

30. Jøranson N, Olsen C, Calogiuri G, Ihlebæk C, Pedersen I. Effects on sleep from group activity with a robotic seal for nursing home residents with dementia: a cluster randomized controlled trial. Int Psychogeriatr. Oct 2021;33(10):1045-1056. [doi: 10.1017/S1041610220001787] [Medline: 32985396]

31. Chen K, Lou VWQ, Tan KCK, Wai MY, Chan LL. Changes in technology acceptance among older people with dementia: the role of social robot engagement. Int J Med Inform. Sep 2020;141:104241. [doi: 10.1016/j.ijmedinf.2020.104241] [Medline: 32739611]

32. van den Berk-Smeekens I, de Korte MWP, van Dongen-Boomsma M, et al. Pivotal response treatment with and without robot-assistance for children with autism: a randomized controlled trial. Eur Child Adolesc Psychiatry. Dec 2022;31(12):1871-1883. [doi: 10.1007/s00787-021-01804-8] [Medline: 34106357]

33. Valentí Soler M, Agüera-Ortiz L, Olazarán Rodríguez J, et al. Social robots in advanced dementia. Front Aging Neurosci. 2015;7:133. [doi: 10.3389/fnagi.2015.00133] [Medline: 26388764]

34. Petersen S, Houston S, Qin H, Tague C, Studley J. The utilization of robotic pets in dementia care. J Alzheimers Dis. 2017;55(2):569-574. [doi: 10.3233/JAD-160703] [Medline: 27716673]

35. Feingold-Polak R, Barzel O, Levy-Tzedek S. A robot goes to rehab: a novel gamified system for long-term stroke rehabilitation using a socially assistive robot-methodology and usability testing. J Neuroeng Rehabil. Jul 28, 2021;18(1):122. [doi: 10.1186/s12984-021-00915-2] [Medline: 34321035]

36. Holeva V, Nikopoulou VA, Lytridis C, et al. Effectiveness of a robot-assisted psychological intervention for children with autism spectrum disorder. J Autism Dev Disord. Feb 2024;54(2):577-593. [doi: 10.1007/s10803-022-05796-5] [Medline: 36331688]

37. Johanson DL, Ahn HS, MacDonald BA, et al. The effect of robot attentional behaviors on user perceptions and behaviors in a simulated health care interaction: randomized controlled trial. J Med Internet Res. Oct 4, 2019;21(10):e13667. [doi: 10.2196/13667] [Medline: 31588904]

38. Kitt ER, Crossman MK, Matijczak A, Burns GB, Kazdin AE. Evaluating the role of a socially assistive robot in children’s mental health care. J Child Fam Stud. 2021;30(7):1722-1735. [doi: 10.1007/s10826-021-01977-5] [Medline: 34025101]

39. Kumazaki H, Yoshikawa Y, Yoshimura Y, et al. The impact of robotic intervention on joint attention in children with autism spectrum disorders. Mol Autism. 2018;9:46. [doi: 10.1186/s13229-018-0230-8] [Medline: 30202508]

40. Logan DE, Breazeal C, Goodwin MS, et al. Social robots for hospitalized children. Pediatrics. Jul 2019;144(1):e20181511. [doi: 10.1542/peds.2018-1511] [Medline: 31243158]

41. Marino F, Chilà P, Sfrazzetto ST, et al. Outcomes of a robot-assisted social-emotional understanding intervention for young children with autism spectrum disorders. J Autism Dev Disord. Jun 2020;50(6):1973-1987. [doi: 10.1007/s10803-019-03953-x] [Medline: 30852783]

42. Papadopoulos C, Castro N, Nigath A, et al. The CARESSES randomised controlled trial: exploring the health-related impact of culturally competent artificial intelligence embedded Into socially assistive robots and tested in older adult care homes. Int J Soc Robot. 2022;14(1):245-256. [doi: 10.1007/s12369-021-00781-x] [Medline: 33907589]

43. Papadopoulou MT, Karageorgiou E, Kechayas P, et al. Efficacy of a robot-assisted intervention in improving learning performance of elementary school children with specific learning disorders. Children (Basel). Jul 31, 2022;9(8):1155. [doi: 10.3390/children9081155] [Medline: 36010046]

44. Robinson H, Broadbent E, MacDonald B. Group sessions with Paro in a nursing home: structure, observations and interviews. Australas J Ageing. Jun 2016;35(2):106-112. [doi: 10.1111/ajag.12199] [Medline: 26059390]

45. Rossi S, Santini SJ, Di Genova D, et al. Using the social robot NAO for emotional support to children at a pediatric emergency department: randomized clinical trial. J Med Internet Res. Jan 13, 2022;24(1):e29656. [doi: 10.2196/29656] [Medline: 34854814]

46. So WC, Wong MKY, Lam CKY, et al. Using a social robot to teach gestural recognition and production in children with autism spectrum disorders. Disabil Rehabil Assist Technol. Aug 2018;13(6):527-539. [doi: 10.1080/17483107.2017.1344886] [Medline: 28673117]

47. Srinivasan SM, Park IK, Neelly LB, Bhat AN. A comparison of the effects of rhythm and robotic interventions on repetitive behaviors and affective states of children with autism spectrum disorder (ASD). Res Autism Spectr Disord. Oct 1, 2015;18:51-63. [doi: 10.1016/j.rasd.2015.07.004] [Medline: 26251668]

48. Stoevesandt D, Jahn P, Watzke S, et al. Comparison of acceptance and knowledge transfer in patient information before an MRI exam administered by humanoid robot versus a tablet computer: a randomized controlled study. Rofo. Aug 2021;193(8):947-954. [doi: 10.1055/a-1382-8482] [Medline: 34111898]

49. Wei CW, Kao HY, Wu WH, Chen CY, Fu HP. The influence of robot-assisted learning system on health literacy and learning perception. Int J Environ Res Public Health. Oct 21, 2021;18(21):11053. [doi: 10.3390/ijerph182111053] [Medline: 34769571]

50. Yun SS, Choi J, Park SK, Bong GY, Yoo H. Social skills training for children with autism spectrum disorder using a robotic behavioral intervention system. Autism Res. Jul 2017;10(7):1306-1323. [doi: 10.1002/aur.1778] [Medline: 28464438]

51. Boumans R, van Meulen F, van Aalst W, et al. Quality of care perceived by older patients and caregivers in integrated care pathways with interviewing assistance from a social robot: noninferiority randomized controlled trial. J Med Internet Res. Sep 9, 2020;22(9):e18787. [doi: 10.2196/18787] [Medline: 32902387]

52. Bradwell H, Edwards KJ, Winnington R, Thill S, Allgar V, Jones RB. Implementing affordable socially assistive pet robots in care homes before and during the COVID-19 pandemic: stratified cluster randomized controlled trial and mixed methods study. JMIR Aging. Aug 24, 2022;5(3):e38864. [doi: 10.2196/38864] [Medline: 35830959]
