# Supplementary material for: Care Robots as Emerging Health Technologies: Systematic Review and Meta-Analysis of Randomized Controlled Trials
Source: J Med Internet Res. 2026 Jun 30;28:e95232. doi: 10.2196/95232 (PMC13318203; doi:10.2196/95232)
Supplement: Checklist 1 [file jmir-v28-e95232-s002.docx]

**PRISMA-S Checklist**

*Care Robots as Emerging Health Technologies: A Systematic Review and Meta-Analysis*

This supplementary file documents adherence to the PRISMA-S extension for reporting literature searches in systematic reviews (Rethlefsen ML, Kirtley S, Waffenschmidt S, et al. PRISMA-S: an extension to the PRISMA Statement for Reporting Literature Searches in Systematic Reviews. Syst Rev. 2021;10(1):39. doi:10.1186/s13643-020-01542-z). All 16 PRISMA-S items are addressed below; full database-specific syntax, line numbers, and per-source record counts are provided in Supplementary File S4.

| **Section/Topic** | **Item #** | **Checklist item** | **Location / Description** | **Reported (Yes/No)** |
| --- | --- | --- | --- | --- |
| **INFORMATION SOURCES AND METHODS** | | | | |
| **Database name** | 1 | Name each individual database searched, stating the platform for each. | S4 Section 2 (and Methods §Information Sources): PubMed/MEDLINE (NLM, https://pubmed.ncbi.nlm.nih.gov/); Embase (Elsevier, https://www.embase.com/); Cochrane CENTRAL (Wiley); CINAHL Complete (EBSCOhost); APA PsycINFO (EBSCOhost). Each database is listed with its provider and interface URL in S4 Section 2 metadata blocks. | **Yes** |
| **Multi-database searching** | 2 | If databases were searched simultaneously on a single platform, state the name of the platform, listing all of the databases searched. | S4 Section 2 (CINAHL Complete and APA PsycINFO blocks): both databases were searched on the EBSCOhost platform but were searched separately, not simultaneously, so each strategy is reported individually with database-specific subject headings (CINAHL Subject Headings; APA Thesaurus terms). | **Yes** |
| **Study registries** | 3 | List any study registries searched. | S4 Section 3 and Methods §Information Sources: ClinicalTrials.gov (https://clinicaltrials.gov/) and the WHO International Clinical Trials Registry Platform (ICTRP, https://trialsearch.who.int/). Search dates and records retrieved are documented in S4 Section 3. | **Yes** |
| **Online resources and browsing** | 4 | Describe any online or print source purposefully searched or browsed (e.g., tables of contents, conference proceedings, web sites), and how this was done. | S4 Section 4: Google Scholar was searched as a supplementary source for grey literature and recent preprints (first 200 records screened by title and abstract; search date 29 April 2026). No formal hand-searching of journal tables of contents or conference proceedings was performed, because (i) the principal journal in this field is indexed in all five primary databases, and (ii) the review's eligibility criteria require RCTs or concurrent-controlled designs, which are uncommon in robotics conference proceedings. | **Yes** |
| **Citation searching** | 5 | Indicate whether cited references or citing references were examined, and describe any methods used for locating cited/citing references. | S4 Section 5: backward citation tracking of the reference lists of all 34 included studies and of four prior systematic reviews on overlapping topics (Pu 2019, Leng 2019, Yu 2022, Scassellati 2012). Forward citation tracking was conducted by submitting the four prior reviews to Google Scholar’s “Cited by” function, with all citing publications screened by title and abstract. No additional eligible studies were identified through citation tracking beyond those already retrieved from the database searches. | **Yes** |
| **Contacts** | 6 | Indicate whether additional studies or data were sought by contacting authors, experts, manufacturers, or others. | S4 Section 6: study authors were contacted only for clarification of reported data (no new studies were identified through author contact). Manufacturers and topic experts were not contacted because the review's eligibility was anchored on peer-reviewed published reports. | **Yes** |
| **Other methods** | 7 | Describe any additional information sources or search methods used. | S4 Section 7: no other methods (e.g., institutional repository searches, dissertation databases, social media monitoring) were used beyond those described in Items 1–6. | **Yes** |
| **SEARCH STRATEGIES** | | | | |
| **Full search strategies** | 8 | Include the search strategies for each database and information source, copied and pasted exactly as run. | S4 Section 2: verbatim, executable, line-numbered syntax provided for all 5 databases (PubMed/MEDLINE, Embase, Cochrane CENTRAL, CINAHL Complete, APA PsycINFO). Trade-name sets and concept blocks (intervention, population, study design) are explicit. Trial registry queries (ClinicalTrials.gov, ICTRP) are reported in S4 Section 3. | **Yes** |
| **Limits and restrictions** | 9 | Specify that no limits were used, or describe any limits or restrictions applied to a search and provide justification. | S4 Section 8 and Methods §Information Sources: no language, date, or publication-type limits were applied at the search stage. At full-text screening, articles for which no English or Korean full text or professional translation was obtainable despite repeated retrieval attempts were excluded (n = 5). An animal-only exclusion (“animals NOT humans”) was applied within PubMed and Embase. | **Yes** |
| **Search filters** | 10 | Indicate whether published search filters were used (as originally designed or modified), and if so, cite the filter(s) used. | S4 Section 9: the Cochrane Highly Sensitive Search Strategy (HSSS) sensitivity-maximizing version (Lefebvre et al. 2022, Cochrane Handbook ch. 4) was used for randomized-trial identification in PubMed/MEDLINE and adapted to Embase syntax. No diagnostic or pediatric filters were used. The CINAHL/PsycINFO RCT term sets were custom-built without a pre-published filter. | **Yes** |
| **Prior work** | 11 | Indicate when search strategies from other literature reviews were adapted or reused for a substantive part or all of the search, citing the previous review(s). | S4 Section 10: the intervention concept block (robot trade names; “social robot”, “socially assistive robot”, “companion robot”, etc.) was informed by Pu et al. 2019, Leng et al. 2019, Yu et al. 2022, and Scassellati et al. 2012. The strategy was substantially expanded with additional trade names (CommU, Kabochan, MiRo, iRobiQ, Joy for All) and synonyms not present in the prior reviews. | **Yes** |
| **Updates** | 12 | Report the methods used to update the search(es) (e.g., rerunning searches, email alerts). | S4 Section 11: the search was rerun in full on 29 April 2026 immediately prior to manuscript revision finalization. Email alerts in PubMed and Google Scholar were maintained between the initial search and the final rerun; new records identified by alerts were screened against the same eligibility criteria. | **Yes** |
| **REPORTING OF SEARCH PROCESS AND OUTPUT** | | | | |
| **Dates of searches** | 13 | For each search strategy, provide the date when the last search occurred. | S4 Section 2 metadata blocks: last search date is 29 April 2026 for all 5 databases and both registries. Hand-searches of journals and conference proceedings were completed by 29 April 2026. | **Yes** |
| **Peer review** | 14 | Describe any search peer review process. | S4 Section 12 and Methods §Search Strategy: the full database-specific syntax was peer-reviewed by a second health-sciences librarian against the PRESS 2015 checklist (McGowan et al. 2016) prior to execution. Iterative refinements were made and documented before final execution. | **Yes** |
| **Total Records** | 15 | Document the total number of records identified from each database and other information sources. | S4 Section 13 and Figure 1 (PRISMA 2020 flow diagram): PubMed/MEDLINE n = 5,935; Embase n = 4,034; Cochrane CENTRAL n = 2,258; CINAHL Complete n = 1,176; APA PsycINFO n = 1; ClinicalTrials.gov n = 75; WHO ICTRP n = 8. Total identified = 13,487 records (databases n = 13,404; trial registries n = 83). Per-database metadata blocks and full yield table are provided in Supplementary File S4 (Sections 2, 3, and 13). | **Yes** |
| **Deduplication** | 16 | Describe the processes and any software used to deduplicate records from multiple database searches. | S4 Section 14 and Methods §Selection Process: records were imported into EndNote 21, automatic deduplication was applied, and remaining duplicates were removed manually within Covidence systematic review software (Veritas Health Innovation). Duplicates removed = 4,990. Unique records taken forward to title/abstract screening = 8,497. | **Yes** |

*From: Rethlefsen ML, Kirtley S, Waffenschmidt S, Ayala AP, Moher D, Page MJ, Koffel JB, on behalf of the PRISMA-S Group. PRISMA-S: an extension to the PRISMA Statement for Reporting Literature Searches in Systematic Reviews. Syst Rev. 2021;10:39. Licensed under CC BY 4.0.*
