# Supplementary material for: Care Robots as Emerging Health Technologies: Systematic Review and Meta-Analysis of Randomized Controlled Trials
Source: J Med Internet Res. 2026 Jun 30;28:e95232. doi: 10.2196/95232 (PMC13318203; doi:10.2196/95232)
Supplement: Checklist 3 [file jmir-v28-e95232-s004.docx]

**PRISMA 2020 Expanded Checklist**

*Care Robots as Emerging Health Technologies: A Systematic Review and Meta-Analysis*

This supplementary file documents adherence to the PRISMA 2020 reporting guideline (Page MJ, McKenzie JE, Bossuyt PM, et al. The PRISMA 2020 statement: an updated guideline for reporting systematic reviews. BMJ 2021;372:n71. doi:10.1136/bmj.n71). For each PRISMA 2020 item, the table below identifies the manuscript section in which the item is addressed.

| **Section/Topic** | **Item #** | **Checklist item** | **Location where reported** | **Reported (Yes/No)** |
| --- | --- | --- | --- | --- |
| **TITLE** | 1 | Identify the report as a systematic review. | Title page; Abstract title; Keywords ('systematic review; meta-analysis'). | **Yes** |
| **ABSTRACT** | 2 | See the PRISMA 2020 for Abstracts checklist (Supplementary File S2). | Abstract; Supplementary File S2. | **Yes** |
| **INTRODUCTION** | 3 | Describe the rationale for the review in the context of existing knowledge. | Introduction (paragraphs 1–3): demographic and workforce drivers; theoretical frameworks (social presence, Kitwood, ASD scaffolding); siloed prior reviews (Pu, Leng, Yu, Scassellati). | **Yes** |
| Objectives | 4 | Provide an explicit statement of the objective(s) or question(s) the review addresses. | Introduction, final paragraph: four prespecified objectives (pooled effects across 7 domains; platform/population/duration moderators; RoB 2 quality; GRADE certainty). | **Yes** |
| **METHODS** | 5 | Specify the inclusion and exclusion criteria for the review and how studies were grouped for the syntheses. | Methods §Eligibility Criteria (PICOS framework; 7 prespecified outcome domains; individually and cluster-randomized trials; explicit exclusions). | **Yes** |
| Information sources | 6 | Specify all databases, registers, websites, organisations, reference lists and other sources searched or consulted, with last-searched date. | Methods §Information Sources: 5 databases (PubMed/MEDLINE, Embase, Cochrane CENTRAL, CINAHL, APA PsycINFO) + 2 trial registries (ClinicalTrials.gov, WHO ICTRP); reference-list and forward-citation tracking; last searched 29 April 2026. | **Yes** |
| Search strategy | 7 | Present the full search strategies for all databases, registers and websites, including any filters and limits used. | Methods §Search Strategy summary; full database-specific syntax provided in Supplementary File S4 (PRISMA-S compliant). | **Yes** |
| Selection process | 8 | Specify the methods used to decide whether a study met inclusion criteria, including reviewer count and independence; details of any automation tools. | Methods §Selection Process: dual independent screening (GK, JJ) in Covidence; κ = 0.89 (title/abstract), 0.92 (full text); third-reviewer adjudication; no AI automation used. | **Yes** |
| Data collection process | 9 | Specify the methods used to collect data from reports, including reviewer count, independence, processes for confirming data; details of any automation tools. | Methods §Data Collection Process and Data Items: piloted form; dual independent extraction; consensus resolution; WebPlotDigitizer 4.6 for graphical data; SD imputation per Cochrane Handbook. | **Yes** |
| Data items | 10a | List and define all outcomes for which data were sought; whether all compatible results were sought, and if not, methods to decide which results to collect. | Methods §Data Items: seven prespecified outcome domains (NPS, QoL, depression, agitation, stress/pain, social-communicative, cognitive); for studies with multiple time points, end-of-intervention values were used; instruments listed per domain. | **Yes** |
|  | 10b | List and define all other variables for which data were sought (participant and intervention characteristics, funding sources); assumptions about missing/unclear information. | Methods §Data Items: study identification, design, country, setting, sample size per arm, mean age, sex distribution, primary diagnosis, robot type/model, session and total intervention duration, facilitator role, control condition, adverse events, dropouts. | **Yes** |
| Study risk of bias assessment | 11 | Specify the methods used to assess risk of bias in the included studies, including tool(s), reviewer count and independence; details of any automation tools. | Methods §Study Risk of Bias Assessment: Cochrane RoB 2 (Sterne 2019) for all 34 RCTs (24 individual + 10 cluster); five domains; dual independent assessment; no automation. ROBINS-I not applied (all 34 are randomized after re-classification). | **Yes** |
| Effect measures | 12 | Specify for each outcome the effect measure(s) used in the synthesis or presentation of results. | Methods §Effect Measures and Synthesis Methods: standardized mean difference (Hedges' g) with 95% CI for all 7 outcomes; sign convention explicit (positive g = favours care robot). | **Yes** |
| Synthesis methods | 13a | Describe the processes used to decide which studies were eligible for each synthesis. | Methods §Synthesis Methods: outcome-domain assignment by instrument-to-domain mapping; cohort-overlap allocation per Senn (2009) so each cohort contributes once per domain. | **Yes** |
|  | 13b | Describe any methods required to prepare the data for presentation or synthesis (e.g., handling of missing summary statistics, data conversions). | Methods §Data Collection Process: SD imputation from 95% CIs/SEs/IQRs per Cochrane Handbook; WebPlotDigitizer extraction for graphical-only data; flagged as Class E–F (text/figure-derived) for sensitivity analysis. | **Yes** |
|  | 13c | Describe any methods used to tabulate or visually display results of individual studies and syntheses. | Methods §Synthesis Methods: forest plots for each domain (Figure 3); contour-enhanced funnel plots for k ≥ 6 domains (Figure 4); per-study Hedges' g and 95% CI in Table 1. | **Yes** |
|  | 13d | Describe any methods used to synthesise results and provide a rationale; if meta-analysis: model, heterogeneity methods, software. | Methods §Synthesis Methods: random-effects meta-analysis; REML estimator for τ²; Hartung–Knapp–Sidik–Jonkman (HKSJ) correction for the pooled CI (rationale: small-sample false-positive control); 95% prediction intervals; R 4.3.2, meta 6.5-0, metafor 4.4-0. | **Yes** |
|  | 13e | Describe any methods used to explore possible causes of heterogeneity (subgroup analysis, meta-regression). | Methods §Synthesis Methods: prespecified subgroup analyses by robot platform (PARO vs humanoid vs other), target population (dementia/older-adult vs pediatric/ASD vs other), and intervention duration (≥10 weeks vs <10 weeks). | **Yes** |
|  | 13f | Describe any sensitivity analyses conducted to assess robustness of the synthesised results. | Methods §Synthesis Methods and Results §Subgroup and Sensitivity Analyses: leave-one-out for the only significant pool (NPS); RoB-restricted (excluding 4 high-RoB studies); data-extraction sensitivity (excluding Petersen 2016); class-restricted (excluding text/figure-derived data). | **Yes** |
| Reporting bias assessment | 14 | Describe any methods used to assess risk of bias due to missing results in a synthesis (reporting biases). | Methods §Reporting Bias Assessment: contour-enhanced funnel plots; Egger's weighted regression test reserved for k ≥ 10 (no domain reached threshold); funnel plots reported for transparency only per Sterne 2011. | **Yes** |
| Certainty assessment | 15 | Describe any methods used to assess certainty (or confidence) in the body of evidence for an outcome. | Methods §Certainty Assessment: GRADE framework (Guyatt 2008) with downgrading for risk of bias, inconsistency, indirectness, imprecision, and reporting bias; produced using GRADEpro GDT; presented in Table 3. | **Yes** |
| **RESULTS** | 16a | Describe the results of the search and selection process, ideally using a flow diagram. | Results §Study Selection: 13,487 records identified; 8,497 unique after deduplication; 121 full-text assessed; 87 excluded; 34 included. Figure 1 (PRISMA 2020 flow diagram). | **Yes** |
|  | 16b | Cite studies that might appear to meet inclusion criteria but were excluded, and explain why. | Results §Study Selection: 87 excluded at full text with explicit reasons (not peer-reviewed n=46; pilot without comparator n=25; non-randomized n=6; no EN/KR translation n=5; no concurrent control n=5). | **Yes** |
| Study characteristics | 17 | Cite each included study and present its characteristics. | Results §Study Characteristics; Table 1 (per-study characteristics for all 34 included RCTs); Table 2 (summary characteristics). | **Yes** |
| Risk of bias in studies | 18 | Present assessments of risk of bias for each included study. | Results §Risk of Bias in Studies; Figure 2 (RoB 2 traffic-light study-level + domain-level summary for all 34 studies). | **Yes** |
| Results of individual studies | 19 | Present, for each study and outcome: summary statistics for each group (where appropriate) and an effect estimate with its precision. | Forest plots (Figure 3) show study-level Hedges' g and 95% CI for every contributing study in every domain; per-study summary stats are available in the data extraction file (Data Availability statement). | **Yes** |
| Results of syntheses | 20a | For each synthesis, briefly summarise the characteristics and risk of bias among contributing studies. | Results §Results of Syntheses (per-domain narrative subsections, e.g., “Neuropsychiatric Symptoms”, “Quality of Life”, etc.). | **Yes** |
|  | 20b | Present results of all statistical syntheses; if meta-analysis, summary estimate, precision, and heterogeneity measures. | Table 3 (Summary of Findings) and Figure 3 (forest plots): pooled HKSJ Hedges' g, 95% CI, 95% prediction interval, I², τ², p-value for each of 7 domains. | **Yes** |
|  | 20c | Present results of all investigations of possible causes of heterogeneity. | Results §Subgroup and Sensitivity Analyses: descriptive subgroup patterns by platform, population, and duration (formal pooling precluded by small per-subgroup k). | **Yes** |
|  | 20d | Present results of all sensitivity analyses conducted. | Results §Subgroup and Sensitivity Analyses: leave-one-out (NPS pool), Petersen-exclusion, RoB-restricted, and class-restricted analyses reported with effect sizes and p-values. | **Yes** |
| Reporting biases | 21 | Present assessments of risk of bias due to missing results (reporting biases) for each synthesis. | Results §Reporting Biases and Small-Study Effects; Figure 4 (contour-enhanced funnel plots for k = 6 NPS and k = 7 depression); Egger's not formally interpreted given k < 10. | **Yes** |
| Certainty of evidence | 22 | Present assessments of certainty (or confidence) in the body of evidence for each outcome. | Results §Certainty of Evidence; Table 3: LOW for NPS; VERY LOW for the remaining six domains; per-domain downgrading rationale documented. | **Yes** |
| **DISCUSSION** | 23a | Provide a general interpretation of the results in the context of other evidence. | Discussion §Principal Findings; §Interpretation in the Context of Prior Reviews (compared with Pu, Leng, Yu, Scassellati). | **Yes** |
|  | 23b | Discuss any limitations of the evidence included in the review. | Discussion §Strengths and Limitations: structural performance bias of unblinded robot trials; heterogeneity (I² = 29–90%); modest sample sizes; data-extraction class limitations. | **Yes** |
|  | 23c | Discuss any limitations of the review processes used. | Discussion §Strengths and Limitations: absence of prospective PROSPERO registration acknowledged; cohort-overlap reclassifications and Petersen 2016 data ambiguity flagged; subgroup formal pooling precluded by small per-subgroup k. | **Yes** |
|  | 23d | Discuss implications of the results for practice, policy, and future research. | Discussion §Targeted Adjunctive Use Cases (three narrow indications); §Clinical and Implementation Implications; §Future Research Directions. | **Yes** |
| **OTHER INFORMATION** | 24a | Provide registration information, including register name and number, or state that the review was not registered. | Front matter (Registration statement) and Methods: “The review was not prospectively registered in PROSPERO.” Acknowledged again in §Strengths and Limitations. | Yes (negative disclosure) |
|  | 24b | Indicate where the review protocol can be accessed, or state that a protocol was not prepared. | Methods: “an a priori review protocol … was developed and dated before the formal search and is available from the corresponding author on reasonable request.” | **Yes** |
|  | 24c | Describe and explain any amendments to information provided at registration or in the protocol. | Not applicable — the review was not prospectively registered. Methodological changes during this revision cycle (HKSJ correction, unified RoB 2, expanded 5-database search) are documented in the response-to-reviewers letter. | N/A (with explanation) |
| Support | 25 | Describe sources of financial or non-financial support, and the role of funders or sponsors. | Funding statement: Keimyung University Dongsan Medical Center research promotion grant (2023). “The funder had no role in study design, data collection, analysis, interpretation, manuscript preparation, or the decision to submit for publication.” | **Yes** |
| Competing interests | 26 | Declare any competing interests of review authors. | Conflicts of Interest statement: “None declared.” | **Yes** |
| Availability of data, code and other materials | 27 | Report which materials are publicly available and where they can be found: data forms; extracted data; data used for analyses; analytic code; other materials. | Data Availability statement: data extraction tables and statistical analysis files available from the corresponding author on reasonable request; primary data extracted from publicly accessible peer-reviewed reports. | **Yes** |

*From: Page MJ, McKenzie JE, Bossuyt PM, Boutron I, Hoffmann TC, Mulrow CD, et al. The PRISMA 2020 statement: an updated guideline for reporting systematic reviews. BMJ. 2021;372:n71. doi:10.1136/bmj.n71. Licensed under CC BY 4.0.*
